# Application of a parametric supermatrix multi-task multimodal deep learning model based on electroencephalogram and peripheral physiological signal fusion in emotion recognition

**DOI:** 10.3389/fnins.2026.1894285

**Published:** 2026-07-31

**Authors:** Xue Li, Piqiang Gong, Chuantao Li, Xiaohan Ding, Fuming Chen

**Affiliations:** 1 Medical Security Center, The 940th Hospital of Joint Logistics Support Force of Chinese People’s Liberation Army, Lanzhou, Gansu, China; 2 Department of Biomedical Engineering, School of Medical Information Engineering, Gansu University of Chinese Medicine, Lanzhou, Gansu, China; 3 Naval Medical Center, Naval Medical University, Shanghai, China

**Keywords:** electroencephalographic signals, emotion recognition, multimodal fusion, multi-task learning, peripheral physiological signals

## Abstract

Accurate emotion recognition under limited computational resources remains challenging in applications such as healthcare, human–computer interaction, and intelligent systems. To address this issue, we propose a parameterized supermatrix multi-task multimodal emotion recognition model (PH-MTM) that fuses electroencephalography (EEG) and peripheral physiological signals (PPS). Physiological signals from the DEAP dataset are preprocessed to extract differential entropy (DE) and power spectral density (PSD) features, which are organized into a “frequency band number × 8 × 9” grid according to the international 10–20 system. An improved squeeze-and-excitation (SE) attention mechanism adaptively assigns weights to different frequency bands, while a convolutional neural network (CNN) performs deep fusion of cross-band and cross-channel information. To reduce model complexity without sacrificing performance, a lightweight fully connected layer based on matrix decomposition is integrated into a multi-task learning framework. Because DEAP includes only 32-channel EEG signals, additional experiments are conducted on the 62-channel SEED dataset to evaluate the scalability and robustness of the frequency-band fusion strategy. PH-MTM achieves 95.99 and 96.51% accuracy for valence and arousal classification on DEAP, and 94.25% accuracy for three-class emotion recognition on SEED. Fusion analysis of EEG with different PPS types shows that EEG combined with electromyography (EMG) performs best, reaching 99.69 and 99.74% accuracy for valence and arousal, respectively. Compared with existing methods, the proposed model demonstrates improved recognition accuracy while maintaining low computational cost. In addition, SHAP visualization is applied to analyze the contribution of different frequency-band features, offering further insight into multimodal physiological signal-based emotion recognition.

## 1 Introduction

Emotions are cognitive processes triggered by external stimuli or personal experiences, manifesting as a psychological state (Gutnik et al., 2006). Emotion recognition is a key area in affective computing, aiming to enable machines to accurately understand human emotions and generate appropriate responses based on existing human-computer interaction knowledge (Afzal et al., 2024). Emotion recognition has a wide range of applications. In addition to healthcare (Islam et al., 2024), human-computer interaction (Chowdary et al., 2023), traffic safety (Zhou et al., 2023), game development (Bravo-Navarro et al., 2025), and education (Salloum et al., 2025), it also plays an important role in military medical research. Some scholars have pointed out that this technology can provide key support for battlefield psychological state monitoring, post-traumatic stress disorder intervention, and psychological assessment in virtual training environments (Perlick et al., 2017; Miao et al., 2018). Existing literature indicates (Martinez-Martin and Fernández-Caballero, 2025; Machová et al., 2023; Kumar and Kumar, 2025; Nandini et al., 2025; Jinnuo et al., 2025) that human emotional states can be effectively identified from human behavior using machine learning and data analysis technologies. However, individuals may sometimes consciously or unconsciously conceal their true emotional states by altering their external behavior, a phenomenon known as social masking (Gori et al., 2021). To address this challenge, many researchers have adopted various neuroimaging and physiological signal assessment techniques to monitor and collect physiological data from the central nervous system (CNS) and peripheral nervous system in real time. These data include EEG, electrooculography (EOG), electromyography (EMG), temperature (Temp), galvanic skin response (GSR), respiratory rate (Resp), and electrocardiography (ECG) (Chen et al., 2026a). Emotion recognition can generally be divided into two categories: single-modal and multi-modal. Due to the complexity of emotions, there are significant differences in physiological responses between individuals. Therefore, emotion recognition models based on a single modality often only provide general results. In addition, the emotion regulation system triggers different neurophysiological responses in different individuals and under different emotional states, posing a significant challenge to single-modal models. Compared to single-modal emotion recognition, emotion recognition models based on multimodal physiological signal fusion have higher robustness and accuracy because they can integrate information from multiple physiological signals. This multimodal fusion method overcomes the limitations of single signals, which cannot fully capture the complexity of emotions, thereby improving the reliability and performance of emotion recognition. Therefore, emotion recognition models based on multimodal physiological signal fusion show broader application prospects in the field of affective computing.

In this work, we propose a new method called Parametric Hypermatrix Multi-Task Multi-Modal (PH-MTM), which integrates the SE attention module, convolutional neural networks (CNNs), and parametric hypermatrix custom fully connected layers into a multi-task learning framework. The contributions of this work can be summarized as follows:

1. PH-MTM solves the challenge of different electrode layouts by introducing a standardized EEG input module encoder. The input module maps EEG features to a unified spatial feature map, ensuring consistency between different electrode layouts.
2. PH-MTM adopts a layered strategy to fuse intra-modal and inter-modal emotional information. In the intra-modal stage, the model combines the spatial topology and statistical features of EEG to extract mode-specific representations, where statistical features are divided into four frequency bands to enhance frequency domain resolution. To maintain structural consistency, other physiological signal channels are mapped according to the EEG topology. In the intermodal stage, a channel attention mechanism is introduced to adaptively adjust the weights of channels and their frequency band features, highlighting key emotional modalities, uncovering complementary information, and improving recognition performance.
3. Compared with traditional emotion recognition models, the PH-MTM model adopts a multi-task learning framework, which makes the learning of emotional dimensions more efficient and simplified. By sharing feature extraction layers, multiple tasks can promote each other, reducing the complexity of model training while avoiding the high computational cost of training independent models for each task.
4. Incorporating the parameterized hypercomplex matrix-based dense layer (PHMDense) into the PH-MTM model can significantly reduce the number of parameters and model complexity while maintaining classification accuracy, thereby optimizing the network architecture, lowering computational resource requirements, enhancing model scalability, and enabling efficient deployment in resource-constrained environments.
5. We used the DEAP dataset for multimodal physiological signal emotion recognition and used the 62-channel EEG from the SEED dataset for emotion recognition experiments to evaluate the adaptability of PH-MTM to different electrode layouts and channel numbers. To verify the robustness of the model, we implemented K-fold cross-validation under subject-dependent experiments to evaluate model stability and used a two-tailed paired t-test to test the significance of the experimental results.
6. Due to the black-box nature of deep learning models, we used the SHAP method to visually analyze the contribution of different frequency bands and features of EEG signals in the DEAP dataset, combined EEG and EMG signals, and EEG signals in the SEED dataset to the emotion classification results.

In summary, the emotion recognition model based on multimodal physiological signals proposed by this research institute not only improves the accuracy of emotion state recognition but also provides new ideas for research in multiple fields. As this model continues to be optimized and developed, it is expected to enable more precise personalized treatment in the future, particularly in the rehabilitation process for patients with emotional disorders, providing more scientific and effective intervention measures to improve patients’ quality of life.

The structure of this paper is as follows: first, we review the latest research work in related fields; then, we outline the architecture of the PH-MTM model and its submodules; next, we introduce the performance evaluation of EEG on different datasets, the visualization of distinguishable feature classification results, ablation experiments, and the performance and combined effects of PH-MTM on different peripheral physiological signals (PPS), and compare its performance with the latest models in recent years; it then presents a visual analysis of the importance of PH-MTM features in EEG and multimodal physiological signals, along with an assessment of computational costs; finally, it summarizes the research findings and discusses potential limitations.

## 2 Related work

In the field of emotion recognition, EEG is considered the gold standard for studying emotional responses. Previous studies have systematically explored EEG-based emotion recognition methods from multiple perspectives, including frequency domain and time domain features, asymmetry in electrical activity between the left and right hemispheres, the role of each brain lobe (frontal lobe, parietal lobe, temporal lobe, occipital lobe) in emotion regulation, and the relationship between frequency bands such as *θ*, *α*, *β*, and *γ* and emotion categories. Additionally, functional brain network modeling has been used to characterize the dynamic connectivity patterns between brain regions under different emotional states. Yuvaraj et al. (2023) proposed an EEG-based emotion recognition method. The system compared the effectiveness of various feature extraction techniques in emotion recognition and emphasized the advantages of fractal dimension features in distinguishing emotional states. Cheng et al. (2020) proposed a method for emotion recognition based on multi-channel EEG using deep forests, utilizing scanning modules to extract spatial features from EEG and cascading forests to model spatiotemporal features and classify emotions. Khare and Bajaj (2020) proposed an EEG-based emotion recognition method that converts signals into images using a smoothed pseudo-Wigner-Ville distribution and classifies them using multiple CNN. Liu et al. (2021) proposed a deep learning model called the three-dimensional convolutional attention neural network, which effectively integrates the features of multi-channel EEG signals through a spatiotemporal feature extraction module and a channel attention mechanism. Li et al. (2018) proposed a Bi-Hemispheric domain adversarial neural network (BiDANN) model that utilizes the differences in emotional responses between the left and right hemispheres of the brain to extract emotional features through adversarial learning. They further proposed BiDANN-S to reduce individual differences and improve the generalization ability of recognition. Guo et al. (2022) proposed a horizontal-vertical feature fusion network (HVF2N-DBR) based on brain regions, which effectively enhanced emotion recognition performance by extracting spatio-temporal features in different directions through brain lobe segmentation and multi-scale convolution. Zhang et al. (2021) proposed a dynamic graph convolutional neural network model with sparse constraints and systematically studied the impact of different EEG frequency band features on emotion recognition. The results showed that the *γ* frequency band features had the strongest emotion discrimination ability. Gao et al. (2023) proposed a robust brain network construction method based on Granger causality analysis. By introducing the student *t*-distribution into the multiple autoregressive model, they effectively suppressed the interference of EOG and other artifacts on EEG, revealing differences in the topological structure of brain networks between men and women under emotional states.

In multimodal emotion recognition research, EEG is typically used as the primary modality due to its high spatio-temporal resolution of CNS emotion processing. PPS (such as ECG, GSR, Resp., and Temp) are used as auxiliary modalities to capture the physiological responses of the autonomic nervous system in different emotional states, thereby achieving more comprehensive and robust emotion recognition. Liu et al. (2023) proposed a multimodal feature weighting method that integrates importance attention and complementarity attention mechanisms, addressing the issues of low interaction utilization of modal information and difficulty in accurately reflecting emotions when conflicts arise in traditional multimodal emotion recognition methods. Ayata et al. (2020) proposed an emotion recognition method based on multimodal physiological signals, extracting features from signals such as Resp., photoplethysmography, and Temp, and using machine learning algorithms such as random forest (RF), support vector machine (SVM), and logistic regression (LR) for emotion state prediction. Wang and Wang (2025) extracted time-domain, frequency-domain, and nonlinear features from EEG and ECG, combined attention mechanisms, one-dimensional convolutional neural networks (1D-CNN), and gated recurrent units (GRU), and performed feature selection using RF to achieve three-dimensional emotion recognition under multimodal fusion. Li et al. (2024) proposed a multimodal physiological signal emotion recognition model to address the issues of inconsistency and fusion redundancy between different physiological signals. The model uses a cross-modal Transformer to optimize auxiliary modal features, employs a low-rank fusion method to reduce redundancy, and enhances primary modal features through an improved cross-modal Transformer. Finally, it fuses multimodal features to achieve emotion recognition. Hou et al. (2023) proposed a multimodal emotion recognition method that integrates discriminant correlation analysis with a time-alignment mechanism. By constructing neural representations of CNS and the autonomic nervous system (ANS), they incorporated emotion labels to enhance the accuracy of conventional correlation analysis and employed Bayesian algorithms to optimize cross-modal time alignment, thereby improving recognition performance and revealing underlying neural dynamic mechanisms. Li and Peng (2024) proposed an end-to-end multimodal emotion recognition method based on facial expressions and non-contact physiological signals. They extracted facial expression features and telemetric photoplethysmography (PPG) signals from facial videos and introduced a cross-modal Transformer attention mechanism to model the correlation between the two modalities. This method has application potential for multimodal emotion recognition in real-world scenarios. Li et al. (2025) proposed an uncertainty-aware graph contrast fusion network (UAGCFNet) to address the issues of intra-modal connection uncertainty and inter-modal semantic deviation in physiological signals. This approach effectively mitigates single-modal bias caused by semantic differences and improves the fusion performance of multi-modal graph representations. Zhu et al. (2025) proposed a multimodal emotion recognition method that uses a dual-stream multi-scale encoder to extract EEG and PPS features, dynamically allocates modal weights through a global–local fusion module, and introduces a Transformer and adaptive collaboration module to optimize intra- and inter-modal correlations, achieving excellent performance on the DEAP and DREAMER datasets. Ramadan et al. (2024) proposed a multimodal emotion recognition method that integrates feature-level and decision-level information, combining EEG, EMG, and EOG to extract statistical features, PSD, and incremental entropy. They used recursive feature elimination (RFE) to screen key features and applied Bagging and K-nearest neighbor algorithms for classification in a combination of the three modalities. Zhang et al. (2020) proposed a regularized deep fusion framework for multimodal physiological signals, which generates dense embeddings through kernel matrices, extracts modal features through deep networks, and introduces regularization terms in the global fusion layer to simultaneously model modal correlations and differences, thereby improving the robustness and generalization ability of emotion recognition.

In recent years, with the continuous advancement of emotion recognition research, multi-task learning has been gradually introduced into this field to simultaneously model prediction tasks for multiple emotion dimensions. Unlike the traditional method of modeling each emotion dimension separately using a single-task model, i.e., the “one dimension, one model, independent prediction” strategy, multi-task learning can achieve parallel prediction of two emotion dimensions in a single model, improving model training efficiency and enhancing feature sharing capabilities between different tasks. This strategy not only reduces model parameter redundancy, but also improves the model’s generalization ability in emotion dimension recognition through a collaborative learning mechanism. Currently, research applying multi-task learning frameworks to emotion recognition has focused primarily on modalities such as speech (Chen et al., 2025), context (Mukherjee, 2025), conversation (Fu et al., 2026), and facial expressions (Xiang et al., 2025), achieving significant progress in these areas. In terms of physiological signals, related research has primarily focused on EEG, demonstrating excellent temporal and spatial resolution advantages in EEG emotion recognition tasks. Li et al. (2022) proposed an EEG emotion recognition method based on multi-task learning, combining capsule networks (CapsNet) and attention mechanisms to simultaneously predict the three dimensions of emotion: arousal, valence, and dominance. In recent work, Dai et al. (2024) proposed a multi-task EEG recognition framework called MTEEG, which combines task-independent time encoders and task-specific low-rank adaptation modules to achieve joint modeling of multiple EEG tasks. This method effectively decouples the parameter space, alleviating task conflicts in multi-task learning, and simultaneously handles tasks such as anomaly detection, event type classification, emotion recognition, epilepsy detection, sleep stage classification, and motor imagery classification without significantly increasing computational and storage costs. It improves training efficiency and model generalization capabilities, demonstrating its potential as a general-purpose brain-computer interface model. Kong et al. (2025) proposed a multi-task residual cross-attention framework for jointly modeling emotion recognition and emotion disorder detection tasks in electroencephalography signals, aiming to explore the synergistic relationship between the two and improve the model’s ability to distinguish between emotional states and emotional disorders and its generalization performance. Li R. et al. (2023) proposed a multi-task EEG emotion recognition model (MTLFuseNet) that fuses spatiotemporal latent features obtained through VAE-based unsupervised learning with time–frequency features extracted via graph convolutional networks (GCN) and gated recurrent units (GRU) under supervised learning, thereby constructing a more discriminative spatiotemporal–spectral fused representation. The model incorporates focal loss to address class imbalance and employs triplet center loss to enhance feature discriminability. Zhu et al. (2024) proposed a multi-source multi-task weight adaptive framework (MS-MWA) based on EEG, which uses a multi-level task-guided model to learn invariant features in the shallow learning domain and unique features in the deep learning domain, thereby improving cross-subject and cross-session generalization capabilities. At the same time, a co-variance uncertainty mechanism is introduced to dynamically adjust the multi-task loss weights, enhancing the robustness and adaptability of the model. However, existing methods are mostly limited to single modalities, and exploration of multimodal fusion of EEG and PPS is still relatively limited. To address this shortcoming, this paper proposes a parametric supermatrix multi-task multimodal emotion recognition model that integrates EEG and PPS, aiming to achieve multi-dimensional feature collaborative modeling and further improve the lightweight design and performance optimization of emotion recognition models.

## 3 Method

**Figure 1:**
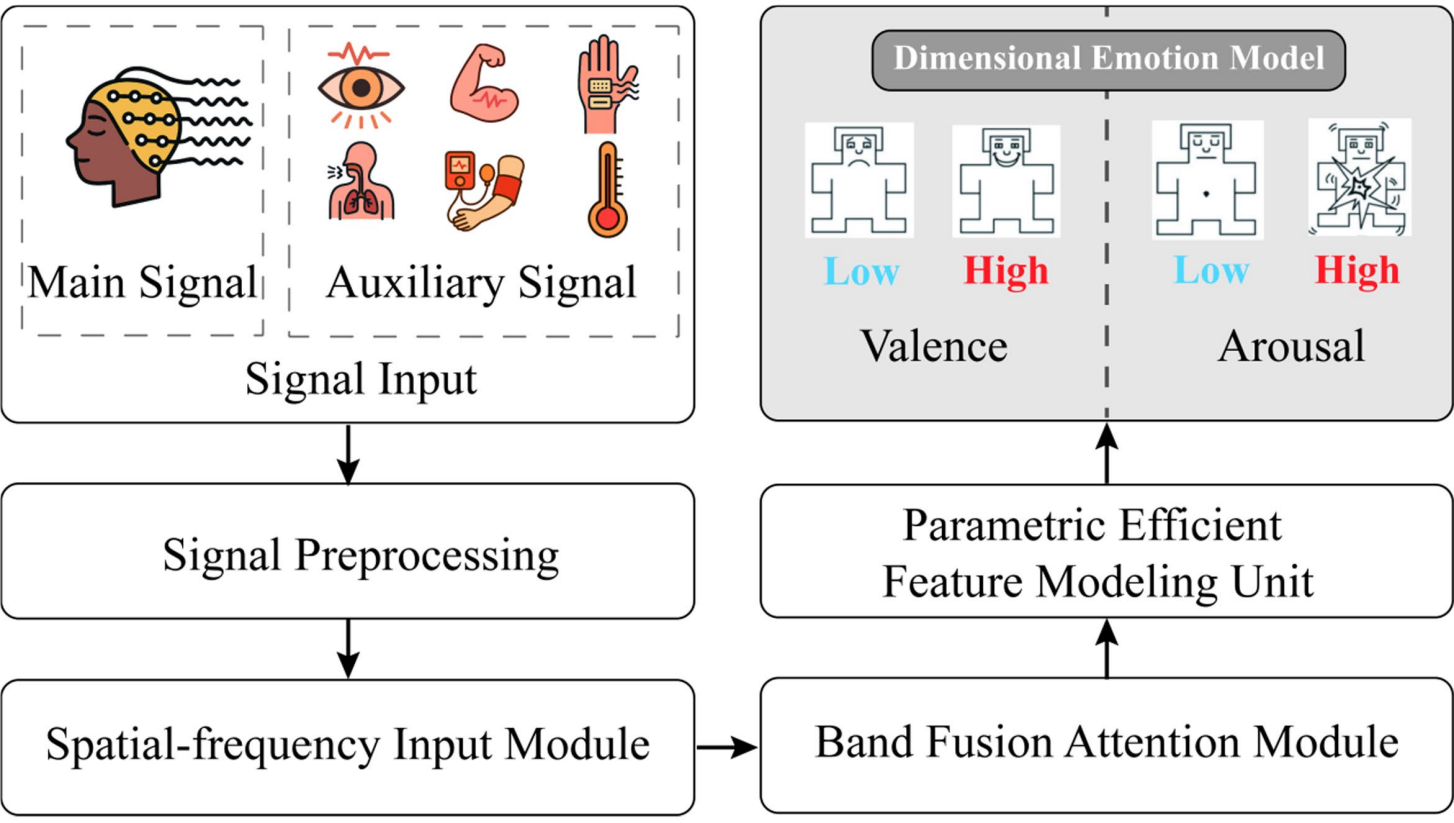
PH-MTM overall architecture.

This study proposes a parameterized hypermatrix multi-task multi-modal emotion recognition model based on the fusion of EEG and PPS, as shown in Figure 1. The overall structure consists of standardized input modules, frequency band fusion attention modules, parameter-efficient feature modeling units, and dual-task classifiers. The model first undergoes pre-processing to extract multi-band features from EEG and PPS, which are then input into a spatial topological alignment input graph. Subsequently, the channel attention mechanism is used to reinforce key modal and frequency band information, and a lightweight parameterized hypermatrix customized fully connected layer is used for feature compression and modeling. Finally, the model uses two parallel softmax branches to simultaneously predict two classification tasks, namely valence and arousal, achieving two-dimensional emotion recognition in a single forward pass. Therefore, this model has good modal fusion, robustness, and computational efficiency.

### 3.1 Signal preprocessing and feature extraction

In the field of emotion recognition, signal preprocessing is a key step in extracting effective information from physiological signals for emotional state classification (Nair et al., 2025). It includes downsampling, baseline correction, data segmentation, filtering, and feature extraction. Before extracting signal features, data is often divided into segments using a 0.5 s time window. This window size is widely used in emotion recognition research (Wang et al., 2024a; Yin et al., 2025) because it accurately captures rapid fluctuations in emotions and effectively suppresses the influence of short-term noise, thereby ensuring the stability and accuracy of signal features. Based on this, the idea of a 0.5 s time window was introduced into the method used in this study.

**Figure 2:**
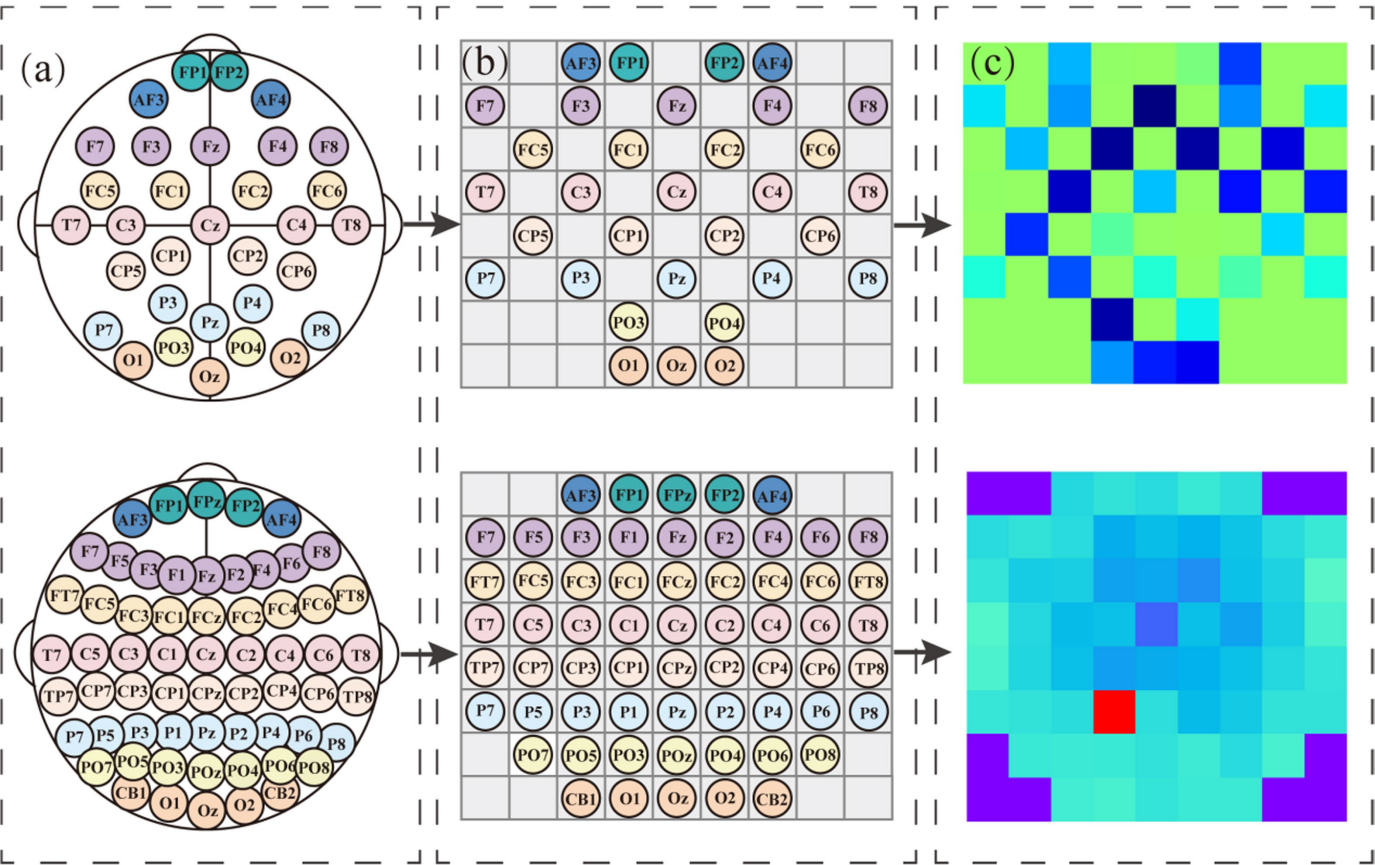
Standardized spatial frequency input module (STIM). **(a)** 2D channel placement. **(b)** Sparse spatial feature matrix. **(c)** Band feature map.

**Figure 3:**
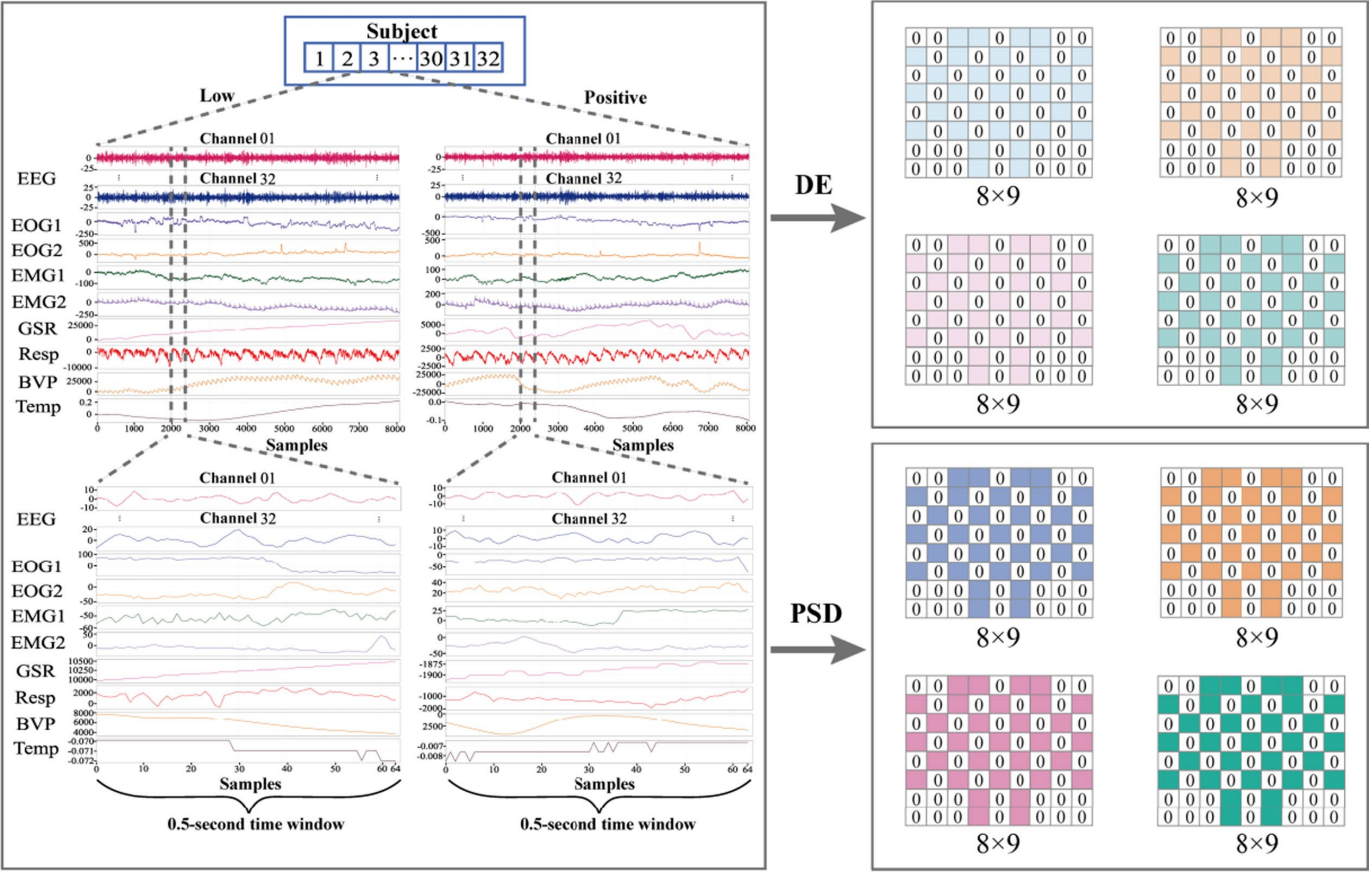
Feature extraction.

The EEG data in the DEAP and SEED datasets have been downsampled and renumbered using channel names. The DEAP dataset includes 3 s of baseline data during the data collection process. The baseline data is divided into six parts using a 0.5 s time window, and then the mean of the six parts is calculated to perform baseline correction on the entire signal, thereby eliminating the impact of baseline drift. The same 0.5 s time window was applied to the experimental data. Then, bandpass filters with frequencies of 4–45 Hz and 0–75 Hz were applied to all EEG channels of the DEAP and SEED datasets, respectively. Regarding PPS in the DEAP dataset, bandpass filters with cutoff frequencies of 0–2.4 Hz, 0–2.4 Hz, 0.01–0.5 Hz, 0–0.2 Hz, 0.5–10 Hz, and 0.5–50 Hz were applied to GSR, RESP, blood volume pulse (BVP), TEMP, EOG, and EMG, respectively. Subsequently, DE and PSD features were extracted for each 0.5 s time window in the baseline data and experimental data sections. DE quantifies the overall activity level of the signal by calculating the signal energy within each time window, reflecting the intensity of the emotional response (Li Q. et al., 2023). PSD uses Fourier transform to reveal the energy distribution of signals in different frequency bands and analyses the relationship between the frequency components of various physiological signals and emotional states (Paul et al., 2025). Both DE and PSD features were extracted for each channel and arranged according to the electrode positions shown in Figure 2b, as shown in Figure 3.

### 3.2 PH-MTM input module

We designed a standardized spatial frequency input module (STIM) to achieve spatial alignment and structural unification of multimodal, multi-band physiological signals, as shown in Figure 2. The first layer extracts frequency domain features from the original multi-channel EEG and PPS, and calculates the DE and PSD features in four frequency bands, respectively. The model extracts four frequency band feature vectors for each physiological signal, ultimately forming a frequency domain feature matrix with dimensions *R*^*C*×*F*^, where *C* represents the number of modes and *F* = 4 represents the number of frequency bands. The second layer maps the above frequency domain features onto a fixed two-dimensional spatial topology grid based on the 10–20 international electrode layout standard, generating a sparse spatial feature tensor with a shape of *R*^8×9^, where 8 and 9 are the number of rows and columns of the spatial matrix, respectively. Figure 2b shows how the 32-channel and 62-channel configurations are mapped to a unified 8 × 9 grid to preserve physiological consistency between channels, with zero values filling in the positions of unmatched channels. PPS also follows an 8 × 9 grid layout, with numerical values filled in the same positions as EEG. Single-channel signals fill each position in the grid with the same value, while dual-channel signals fill positions alternately to maintain structural consistency. The third layer combines the feature representations of all frequency bands to generate a unified shape tensor (*R*^*F*×8×9^), which is used as input for the model’s main network.

### 3.3 Bandwidth fusion attention mechanism module

In this work, we apply the standard Squeeze-and-Excitation (SE) module to different frequency bands of 8 × 9 matrices obtained from different physiological signals DE and PSD, and perform adaptive weighting of the features of each frequency band, which is called the band fusion attention mechanism (BFAM). This module uses weighted processing for each frequency band, enabling the model to more accurately capture differences and correlations between frequency bands in different modalities, thereby further enhancing the model’s perception and representation capabilities. Let the input signal tensor *X* ∈ *R*^*H*×*W*×*C*^, where *H* is the height of the input matrix, *W* is the width of the matrix, and *C* represents the number of frequency bands. Each signal is characterized as *X*_*h*,*w*,*c*_ (where *h* and *w* represent the height and width of the matrix, respectively, and *c* is the number of frequency bands), and the statistic *s_c_* is obtained through global average pooling, as shown in Equation 1:


$$s_{c}=\frac{1}{H\times W}\sum_{h=1}^{H}\sum_{w=1}^{W}X_{h,w,c}$$ (1)

Let *s* = [*s*_1_, *s*_2_, …, *s_c_*]*^T^*. Here, *s_c_* is the global average pooling value of the *c*th frequency band. Then, all *s_c_* form a vector *s* ∈ *R^c^*.

On this basis, the SE attention module is used to adaptively model the importance of each modality and frequency band. Specifically, the statistics *s_c_* of each frequency band first pass through two fully connected layers, as shown in Equations 2 and 3:


$$z=\text{ReLU}(W_{1}s+b_{1})$$ (2)

$$\alpha =\sigma (W_{2}z+b_{2})$$ (3)

Among these, *W*_1_ ∈ *R*^*C*/*r*×*C*^ and *W*_2_ ∈ *R*^*C*×*C*/*r*^ are the weight matrices for compression and restoration, respectively, *r* is the compression ratio, ReLu (·) is the ReLU activation function, *σ* (·) is the Sigmoid activation function, and *α* = [*α*_1_, *α*_2_, …, *α_c_*]*^T^* are the weighted coefficients for all frequency bands, satisfying *α_c_* ∈ [0, 1].

Finally, by weighting and merging the original features, a weighted feature map $\tilde{X}$ is obtained, calculated using Equation 4:


$${\tilde{X}}_{h,w,c}=\alpha_{c}\cdot X_{h,w,c}$$ (4)

**Figure 4:**
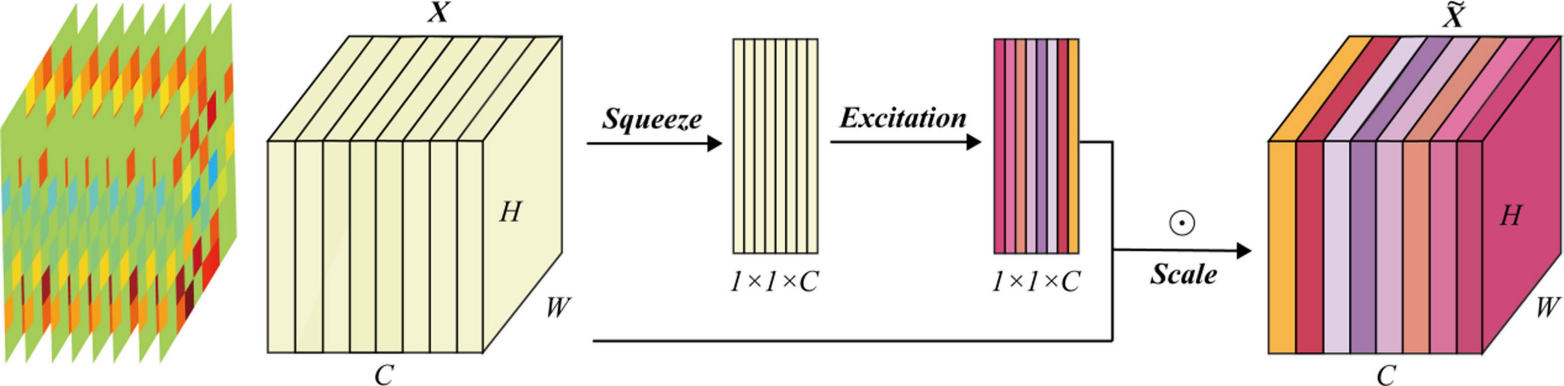
BFAM module architecture.

BFAM uses weighted fusion to effectively strengthen the feature contribution of frequency bands that are highly correlated with the emotional dimension, while suppressing redundant and noisy information. The resulting fusion feature map retains key information across modalities and frequency bands, providing high-quality input for subsequent feature extraction. The module architecture is shown in Figure 4.

### 3.4 Parameter-efficient feature modeling unit

**Figure 5:**
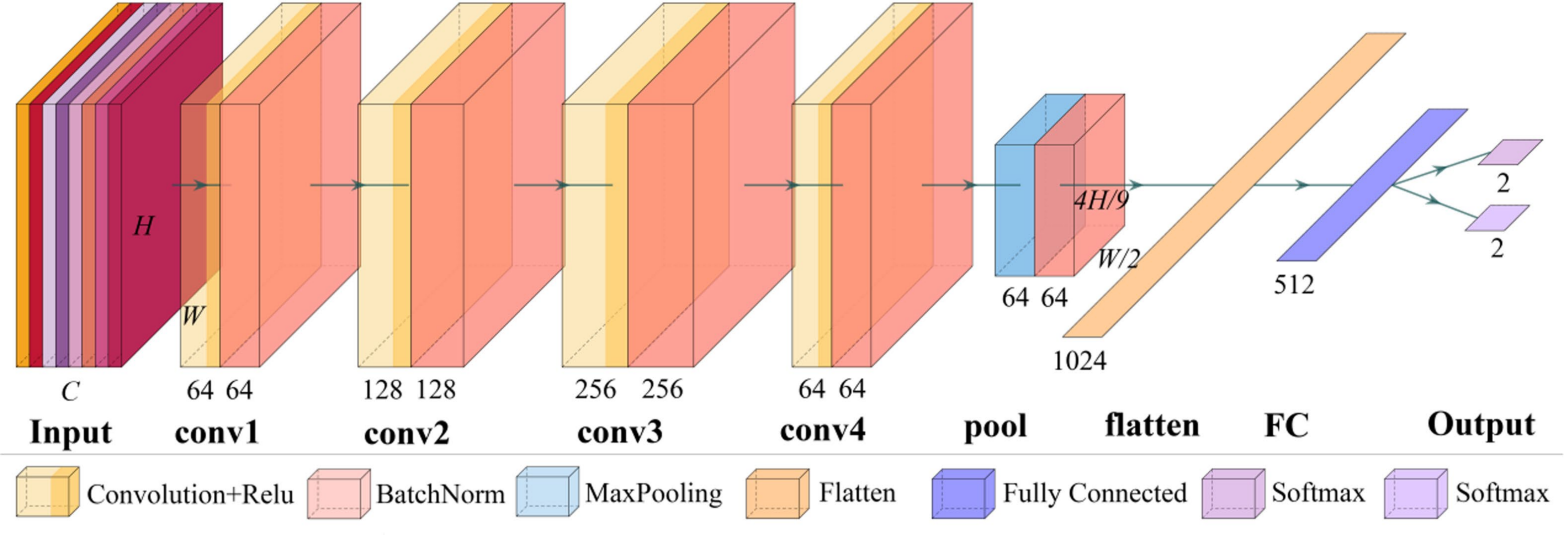
Parameter-efficient feature modeling unit architecture.

To improve the expressive power and operational efficiency of physiological signal emotion recognition models, this paper proposes a parametric efficient feature modeling unit (PEF) centered on a multi-layer CNN, a parameterized hypermatrix custom fully connected layer (PHMDense), and a multi-task learning branch. Its overall structure is shown in Figure 5.

The hyperparameters in Table 1 were selected based on comprehensive prior knowledge, dataset characteristics, and common practices in existing literature. In terms of setting hyperparameters such as the number of convolutional kernels, output dimension, maximum training iterations, batch size, and initial learning rate, this study conducted reasonable optimization based on previous literature experience and model structure characteristics to ensure the comparability of experimental results across different datasets.

**Table 1:** Hyperparameter configuration used to train PEF on each dataset.

Dataset	DEAP	SEED
Number of physiological signal frequency bands	8	10
PEF-1st core number	64	64
PEF-2nd core number	128	128
PEF-3rd core count	256	256
PEF-4th core number	64	64
Output dimension	512	512
Maximum number of training rounds	400	400
Batch size	64	64
Initial learning rate	0.001	0.001
Optimizer	Adam	Adam
Loss function	Categorical_crossentrop	Categorical_crossentrop

#### 3.4.1 PHMDense layer

In traditional neural networks, the fully connected layer (Dense layer) is one of the most widely used basic layers. This layer performs a linear transformation on the features by multiplying the input features by the weight matrix. However, as the network size increases, the fully connected layer introduces a large number of parameters, leading to a sharp increase in computational complexity. Therefore, many researchers have focused on optimizing the Dense layer to reduce the number of parameters and computational complexity while maintaining or improving model performance. The Fire module in SqueezeNet proposed by Iandola et al. (2016) reduces the computational load by reducing the parameters of convolution operations. EfficientNet, proposed by Tan (2019), optimizes the depth, width, and resolution of the network through a composite scaling strategy, thereby reducing the computational burden. MobileNet, proposed by Howard et al. (2017), significantly reduces the amount of convolution computation through depth-separable convolution, making it particularly suitable for low-resource devices. However, these methods still face challenges in terms of insufficient feature extraction capabilities and computational efficiency. To address this issue, this paper introduces the concept of non-negative matrix factorization (NMF) from linear algebra, decomposing the weight matrix in the traditional Dense layer into two smaller tensors, A and S, and then performing element-wise multiplication operations for modeling, thereby reducing the number of parameters and improving computational efficiency.

##### 3.4.1.1 Specific implementation process

Let the input tensor be *X* ∈ *R*^*L*×*I*^, where *L* is the number of samples and *I* is the dimension of the input features. The output tensor of the PHMDense layer is *Y* ∈ *R*^*L*×*O*^, where *O* is the dimension of the output features, and the final weight dimension is *O* × *I*.

Specifically, the PHMDense layer first divides the input and output feature dimensions into blocks according to the hyperparameter *n*. Here, *n* is equivalent to the “rank” in NFM, but it can only take values such as 2, 4, 6, 8, etc., which are even numbers divisible by the number of input and output features. The supermatrix *A* ∈ *R*^*n*×*n*×*n*^ is used to model the interrelationships among input features, while the structure matrix *S* ∈ *R*^*n*×*O*/*n*×*I*/*n*^ is used to partition input and output features, as shown in Equation 5:


$$W=\sum_{k=0}^{n-1}A_{k}⊗S_{k}$$ (5)

By multiplying the input tensor *X* ∈ *R*^*L*×*I*^ with the constructed weight matrix *W*∈*R*^*O*×*I*^, the output *Y* ∈ *R*^*L*×*O*^ is obtained. That is Equation 6:


$$Y=X\cdot W^{T}+b$$ (6)

Among them, *b* is the bias term, which is usually randomized to optimize the training process.

The ReLU activation function is applied to the output of the PHMDense layer to enhance the model’s nonlinear representation capabilities. The activated output is shown in Equation 7:


$$Y_{\text{activated}}=\sigma (Y)$$ (7)

In this context, *σ* (·) denotes the activation function.

##### 3.4.1.2 Parameter analysis and reduction mechanism

In traditional dense layers, the number of parameters is given by Equation 8:


$$P_{\text{Dense}}=I\times O+O$$ (8)

However, in the PHMDense layer, the number of parameters is reduced through matrix decomposition. Assuming that the dimension of the supermatrix *A* is *n* × *n* × *n*, and that we divide the input features and output features into n sub-blocks (i.e., *I*/*n* and *O*/*n*), then the number of parameters in the PHMDense layer is given by Equation 9:


$$P_{\text{PHMDense}}=n\times n\times n+n\times \frac{O}{n}\times \frac{I}{n}+O=n^{3}+\frac{\mathrm{OI}}{n}+O$$ (9)

In practical applications, *n* is usually a small constant. Through this compression mechanism, the number of parameters in the PHMDense layer is usually lower than that in the traditional Dense layer.

#### 3.4.2 Multitasking

**Figure 6:**
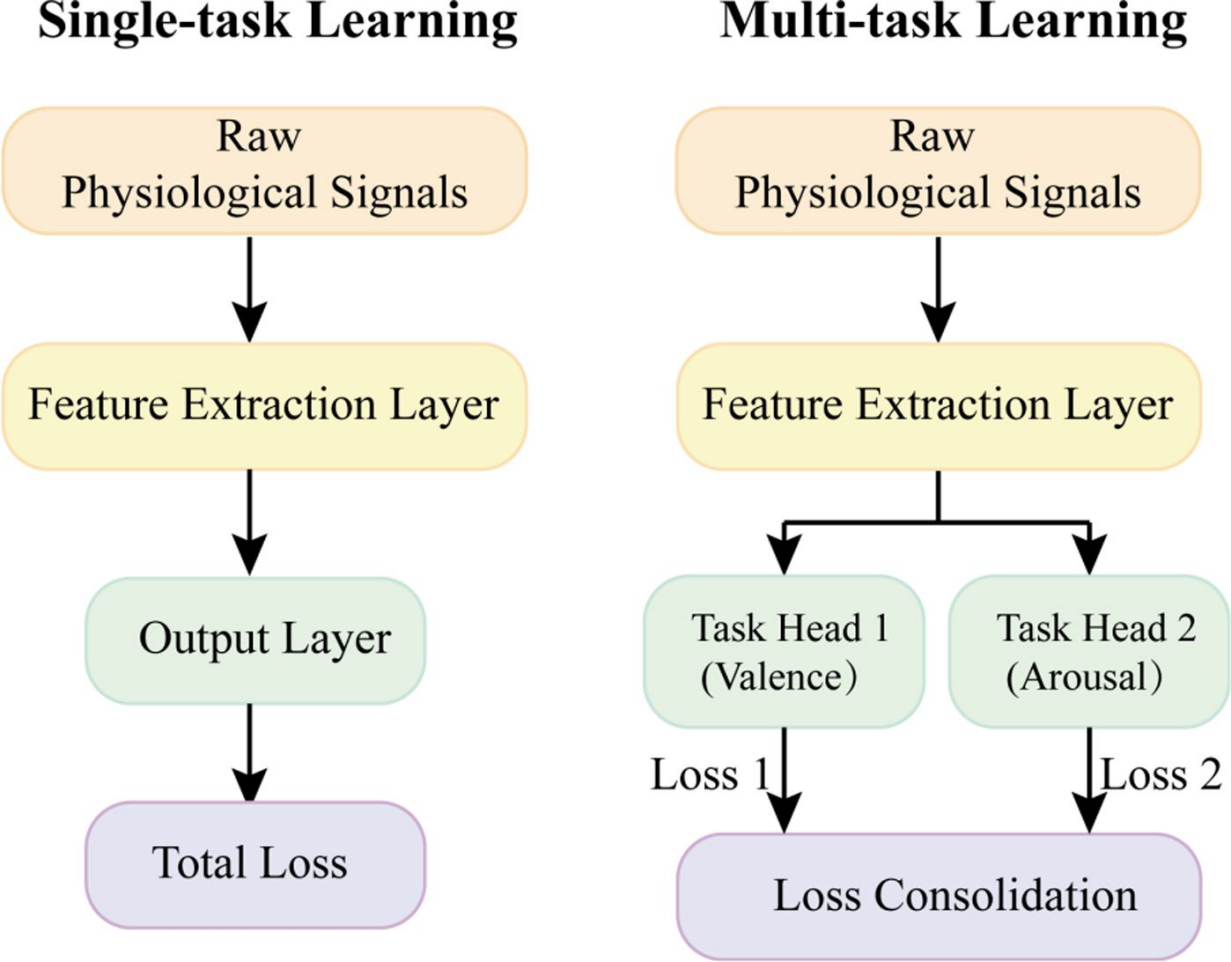
Single-task learning and multi-task learning.

In emotion recognition research based on physiological signals, it is usually necessary to predict the two major dimensions of emotion: valence and arousal. Since both classification tasks use the same physiological signals as input, if traditional single-task learning methods are used, independent models must be trained for each task. This not only causes redundancy in feature extraction and model deployment, but also makes it difficult to uncover the potential connections between them. In contrast, multi-task learning can achieve joint modeling of Valence and Arousal in a single training process by utilizing shared inputs and features, thereby effectively improving the efficiency of the model. A structural comparison between single-task learning and multi-task learning is shown in Figure 6.

This study introduces a hard parameter sharing mechanism from multi-task learning into the model. The model first uses four layers of “convolution-normalization-activation” units to extract features from the input data step by step, and effectively captures local spatial structure information by stacking four sets of convolution modules. Subsequently, feature maps are downsampled and standardized through pooling and normalization operations to enhance feature stability and expressiveness. Next, the network flattens the extracted high-dimensional features and inputs them into the PHMDense layer to obtain a more discriminative global representation. On this basis, the model sets task branches after the PHMDense layer to make predictions for the valence and arousal classification targets, respectively, achieving end-to-end joint optimization under a multi-task learning framework.

From an algorithmic perspective, multi-task learning enables the model to automatically capture the correlation between valence and arousal tasks in a shared representation space by jointly optimizing the loss functions of two related tasks. On the one hand, this helps improve the model’s overall ability to recognize emotional dimensions; on the other hand, it also reduces the risk of the model overfitting to a single task. The overall loss function is designed as a weighted sum of two task losses, and all shared and task-specific parameters are optimized simultaneously through backpropagation to achieve collaborative learning for both objectives.

### 3.5 SHAP visualization method

Given the black-box nature of deep learning algorithms, this study introduces the SHAP method to improve model interpretability. Its core idea is based on the Shapley value in cooperative game theory, which measures the marginal contribution of a single frequency band to the model prediction. The input feature of this model is a tensor of “frequency band × 8 × 9,” where 8 × 9 is the spatial position dimension. Use GradientExplainer to calculate the SHAP value for each spatial position of each frequency band, as shown in Equation 10:


$$\phi_{i,j_{1},j_{2}}=E_{z\in B}[\int_{0}^{1}\frac{\partial f_{k}(z+\alpha (x-z))}{\partial z_{j}}d\alpha (x_{j}-z_{j})]$$ (10)

where *ϕ*_*i*,*j*1,*j*2_ is the SHAP value of frequency band *i* at spatial position ( $j_{1}$ , $j_{2}$ ), *f_k_* is the model prediction function, *x_j_* is the *j*th feature (corresponding to spatial position $j_{1}$ , $j_{2}$ in the input tensor), *z* is the reference sample selected from the background samples, and *α* is the weighting coefficient used for integration calculations. Then, the SHAP values for each feature are aggregated by frequency band to obtain the contribution of each frequency band to the final classification result. As shown in Equation 11:


$$\phi_{i}^{\mathrm{avg}}=\frac{1}{8\times 9}\sum_{j_{1}=1}^{8}\sum_{j_{2}=1}^{9}\phi_{i,j_{1},j_{2}}$$ (11)

Among them, *ϕ_i_* is the SHAP value of the *i*-th frequency band. Through cross-sample aggregation, the absolute values of the SHAP values of different frequency bands in each sample are summed and averaged to obtain the global importance of each frequency band. See Equation 12:


$$\bar{|\mathrm{\phi i}|}=\frac{1}{N}\sum_{i=1}^{N}|\phi_{i}|$$ (12)

Among them, *N* represents all test set samples. Finally, the results of different frequency bands are sorted in descending order to obtain the global contribution ranking of different frequency bands.

### 3.6 Algorithm

Algorithm 1 provides the complete pseudocode for the algorithms related to the PH-MTM model. The process is concise, clear, and easy to reproduce.

Algorithm 1 Parametric hyper-matrix multi-task multi-modal (PH-MTM)
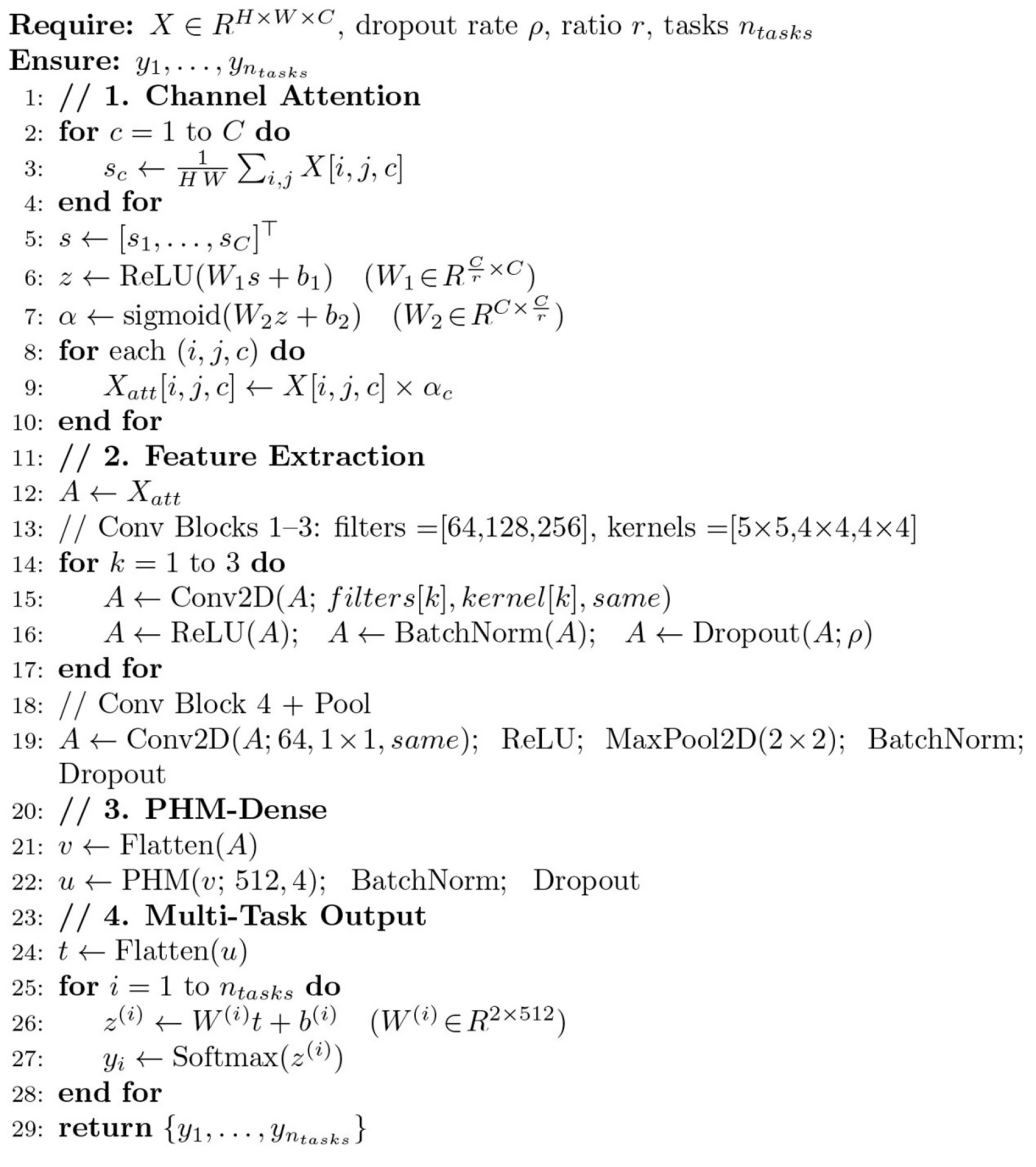

## 4 Experiments and results

### 4.1 Experimental setup

The experimental platform configuration for this study comprises an Nvidia RTX 4090D graphics card (24 GB VRAM) paired with an AMD EPYC 9754 processor featuring 15 vCPUs and 128 cores. The software environment utilized Python 3.8 (running on Ubuntu 20.04), with the deep learning framework TensorFlow 2.9.0 and CUDA version 11.2. All model training and evaluation were conducted within this hardware and software environment. In the experimental design, the dataset was split into training and test sets at a 0.8:0.2 ratio at the trial level. Taking the DEAP dataset as an example, this involved aggregating the experimental data from 32 subjects (40 trials per subject) into 1,280 trials, each assigned a unique trial identifier (ID). Each trial comprised 120 samples (each sample representing a 0.5 s time window within the 60 s trial duration), with all 120 samples sharing identical IDs. For instance, all 120 samples in Trial 0 bore ID 0, while those in Trial 1 bore ID 1. This partitioning ensures that data from the same experiment for a single subject appears only once in either the training or test set, thereby preventing temporal leakage. The training set is further divided into sub-training and validation sets using five-fold cross-validation at the trial level (Chen et al., 2026b,c). The sub-training set is used for model training, while the validation set serves model selection and hyperparameter optimization. An independent test set is employed for the final evaluation of model performance.

### 4.2 Dataset used for validation

To validate the performance of the PH-MTM model, we employed two publicly available datasets: DEAP and SEED. The DEAP dataset served as the primary training dataset, while the SEED dataset was utilized solely to verify the efficacy of the EEG 62-channel frequency band fusion method. As the SEED dataset inherently carries tri-class labels and encompasses only a single-classification task, we accordingly adjusted the PH-MTM model by removing its multi-task branch. The DEAP dataset was jointly released by Queen Mary University of London, University of Twente, University of Geneva, and Swiss Federal Institute of Technology, and includes EEG and various physiological signals (such as GSR, ECG, EOG, etc.) from 32 subjects watching 40 60 s music videos. Before each video is played, 3 s of resting baseline data is recorded. All signals are downsampled to 128 Hz and passband filtered. After watching the video, participants rated their emotional state based on four dimensions (1–9 points): pleasantness, arousal, dominance, and liking. The SEED dataset was provided by the Brain Computing Science Laboratory at Shanghai Jiao Tong University. In the experiment, 15 Chinese subjects watched 15 Chinese movie clips as emotional stimuli, covering positive, neutral, and negative emotions, each lasting about 4 min. Each subject participated in three experiments, with a one-week interval between experiments, resulting in a total of 45 experimental data files. The data set has been downsampled to 200 Hz and preprocessed with bandpass filtering over different ranges. Table 2 lists the key parameters of the DEAP dataset and the SEED dataset.

**Table 2:** Description of two datasets and preprocessing parameters.

Dataset	DEAP	SEED
Emotional stimulus	Music video	Film editing
Subject	32	15
Experiment	40	3
EEG channel	32	62
Peripheral physiological signal channel	8	3
Sampling rate	512 Hz	1,000 Hz
Downsampling rate	128 Hz	200 Hz
Slice window size	0.5 s	0.5 s
Baseline time	3 s	-
EEG filter passband	4–45 Hz	0–75 Hz
Experimental time	60s	240 s
Total sample size	153,600	305,460

### 4.3 Performance of PH-MTM on multiple EEG datasets

#### 4.3.1 DEAP dataset

**Figure 7:**
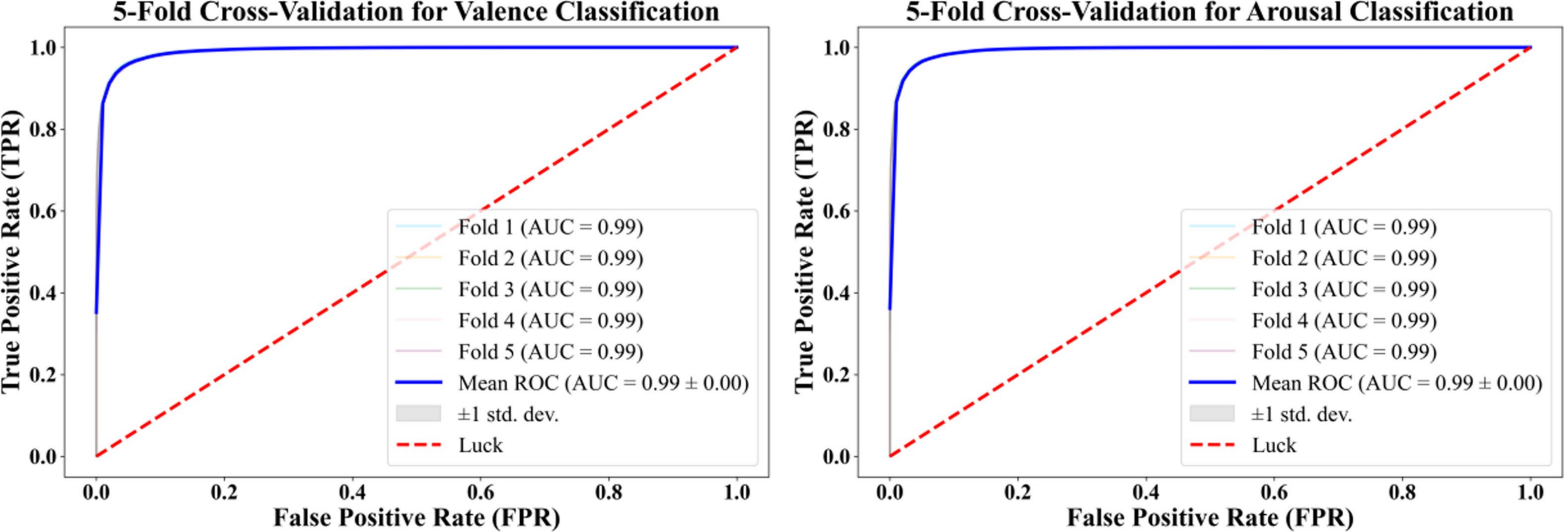
ROC curve for the valence and arousal binary classification task.

When performing binary emotion classification of Valence and Arousal on the DEAP dataset, our method achieved excellent discrimination performance in five-fold cross-validation (as shown in Figure 7). From the ROC curves of each fold, which are almost tightly packed in the upper left corner, and the AUC of each fold, which is 0.99, it can be seen that regardless of the data division threshold selected, the model can correctly distinguish between the two types of emotional states with a probability of approximately 99%. Even more encouraging is that the ±1 STD gray band around the average ROC (blue line) is very narrow, indicating that overall performance is not only excellent but also highly consistent across different data partitions. Compared to random diagonal guessing, our method achieves a true positive rate of over 90% with an extremely low false positive rate, perfectly balancing sensitivity and specificity. In summary, these results fully demonstrate the near-perfect discriminative ability and excellent robustness of our model on the DEAP dataset benchmark.

**Figure 8:**
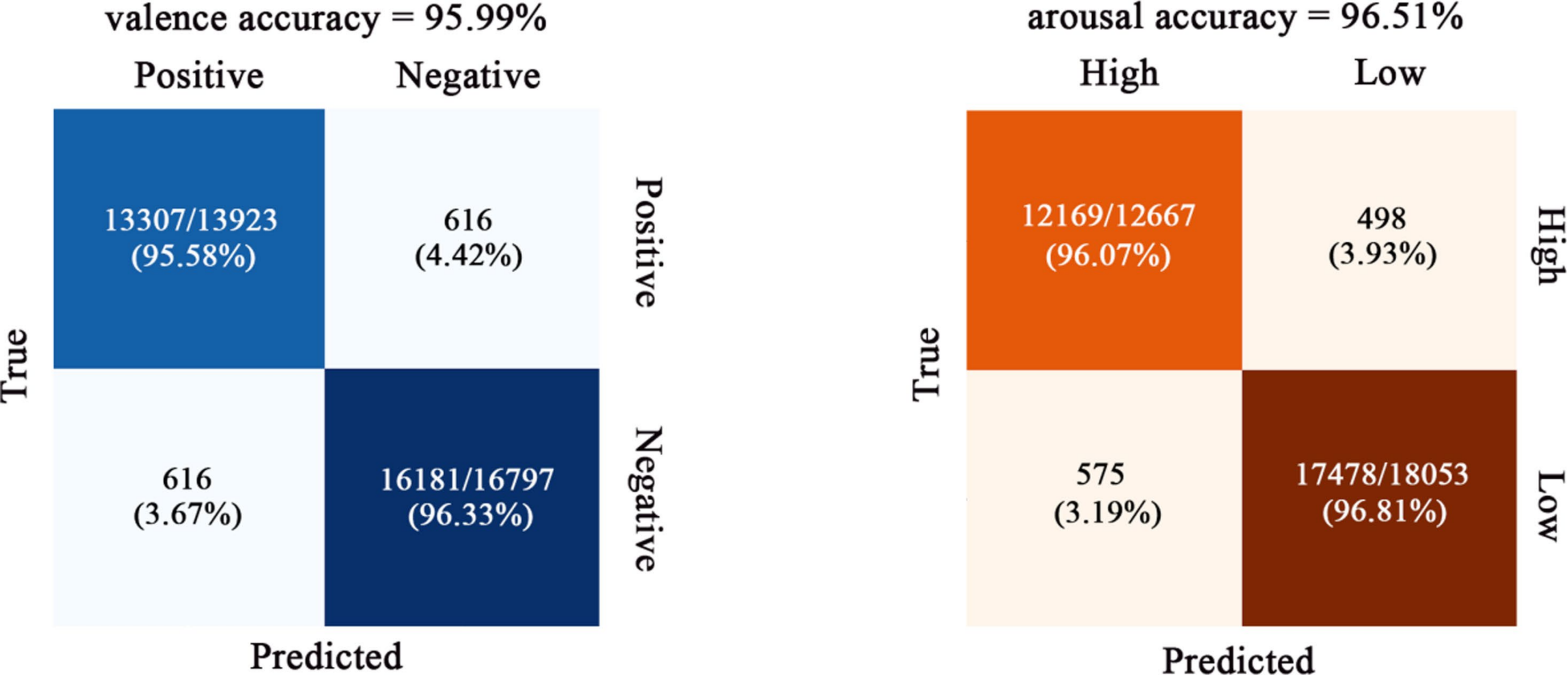
Confusion matrix for the valence and arousal binary classification task.

**Figure 9:**
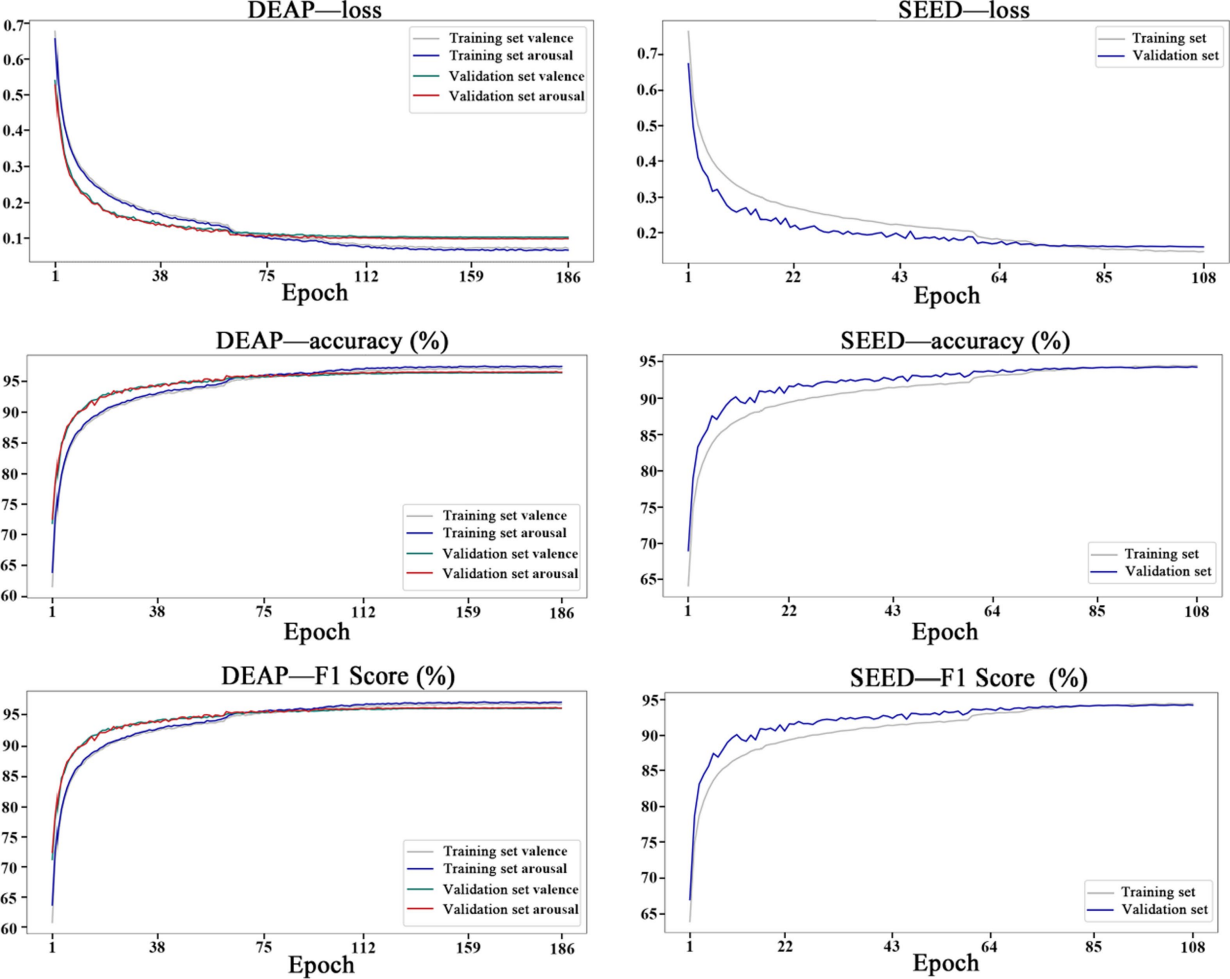
Loss, accuracy, and *F*1-score curves for the best models on the DEAP and SEED datasets.

On this basis, the confusion matrix further confirms that the model has excellent stability and high accuracy in the valence and arousal binary classification task, as shown in Figure 8. On the test set, the valence task achieved an overall accuracy of 95.99% with an *F*1-score of 96.51%; recall for the valence categories stood at 95.58% (13,307/13,923) and 96.33% (16,181/16,797) respectively. For the arousal task, accuracy was 96.51% with an *F*1-score of 96.40%; recall for arousal categories was 96.07% (12,169/12,667) and 96.81% (17,478/18,053). The proportion of misclassified samples remained below 5% across all categories, demonstrating strong consistency with the aforementioned ROC curve results (average AUC = 0.99). The proportion of misclassified samples was less than 5% in all cases, which was highly consistent with the previous ROC curve results (average AUC = 0.99). Additionally, the left column of Figure 9 shows the loss function, accuracy, and *F*1-score curves for the training set and validation set during the final fold of training.

In addition to the DEAP dataset, which contains 32-channel EEG physiological signals, we also introduced a 62-channel EEG dataset, the SEED dataset, to evaluate the adaptability of PH-MTM to different electrode layouts and channel numbers.

#### 4.3.2 SEED dataset

**Figure 10:**
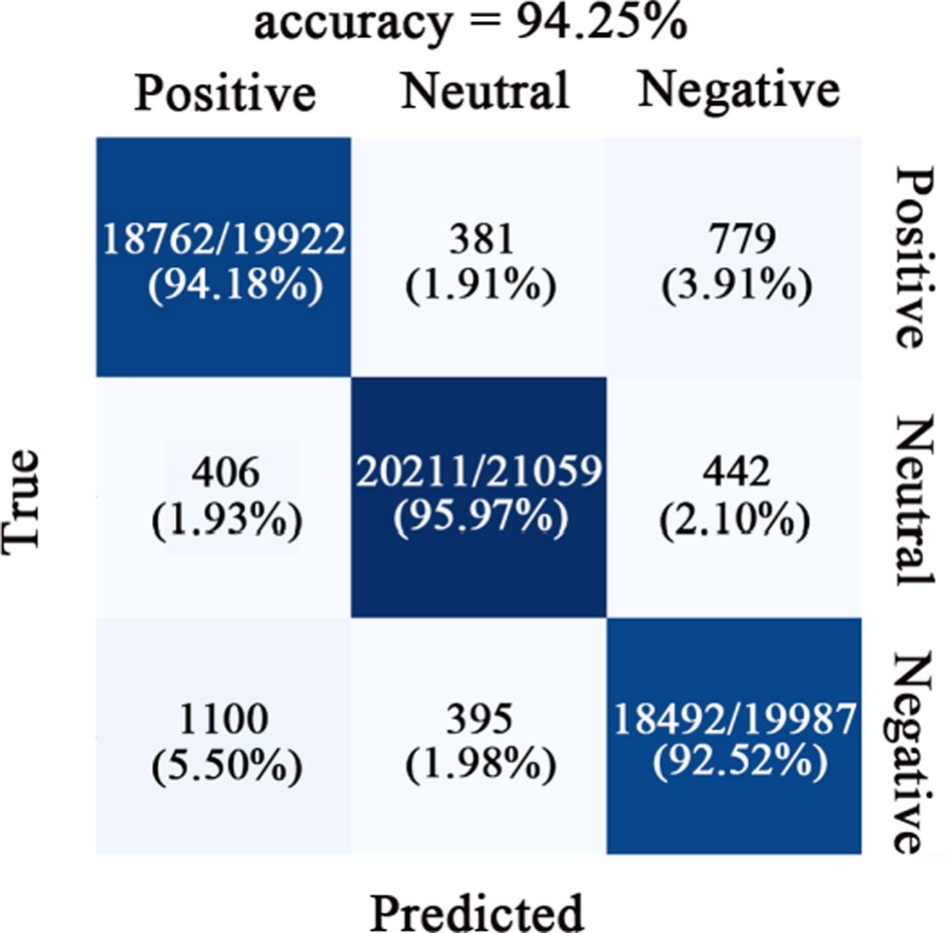
Confusion matrix for the three-classification task.

To assess the effectiveness of the model in extracting features and its adaptability to different spatial electrode layouts and channel numbers, we extracted five frequency bands (*δ* (1–4 Hz), *θ* (4–8 Hz), *α* (8–14 Hz), *β* (14–31 Hz), and *γ* (31–51 Hz)) from the SEED dataset (which differs from the four frequency bands extracted from the DEAP dataset). Figure 10 shows the confusion matrix results for the three-classification (positive, neutral, negative) task based on this dataset.

As evident from the confusion matrix in Figure 10, the model demonstrates high accuracy across all categories (positive, neutral, negative) in the SEED dataset, with a relatively low number of misclassified samples. The model achieves an accuracy of 94.25%, indicating its ability to correctly classify the majority of samples. Recall rates for each category were 94.18% (18,762/19,922), 95.97% (20,211/21,059), and 92.52% (18,492/19,987), respectively. This indicates the model exhibits good balance during classification, with consistent performance across different categories. The model’s *F*1-score of 94.22% further demonstrates the equilibrium between precision and recall. Experimental results indicate that training a three-classification model using DE features from different frequency bands as input yields equally favorable outcomes for 62-channel EEG data. Figure 9: The right-hand column displays the loss, accuracy, and *F*1-score curves for the training and validation sets of the optimal model on the SEED dataset under five-fold cross-validation. The left-hand column shows the three curves for the DEAP dataset, while the right-hand column presents the three curves for the SEED dataset.

As shown in Figure 9, the training and validation curves for the sentiment classification task on the DEAP dataset demonstrate a rapid decline in loss alongside concurrent improvements in accuracy and *F*1-score during the initial 38 epochs. Subsequently, the curves stabilize around 75 epochs, exhibiting high overlap between training and validation metrics. Ultimately, both training and validation accuracy stabilized above 95%, with an *F*1-score exceeding 95%. No significant overfitting on the validation set was observed, demonstrating the model’s rapid convergence and high classification capability. As the SEED dataset was solely employed to validate the effectiveness of feature extraction and assess the model’s adaptability to varying electrode layouts and channel counts, the core experimental section of this study (Sections 4.5 to 4.7) will uniformly utilize the DEAP dataset for result analysis. This will be consistently applied throughout the subsequent discussion without further specification.

### 4.4 Visualization of EEG distinguishability features

**Figure 11:**
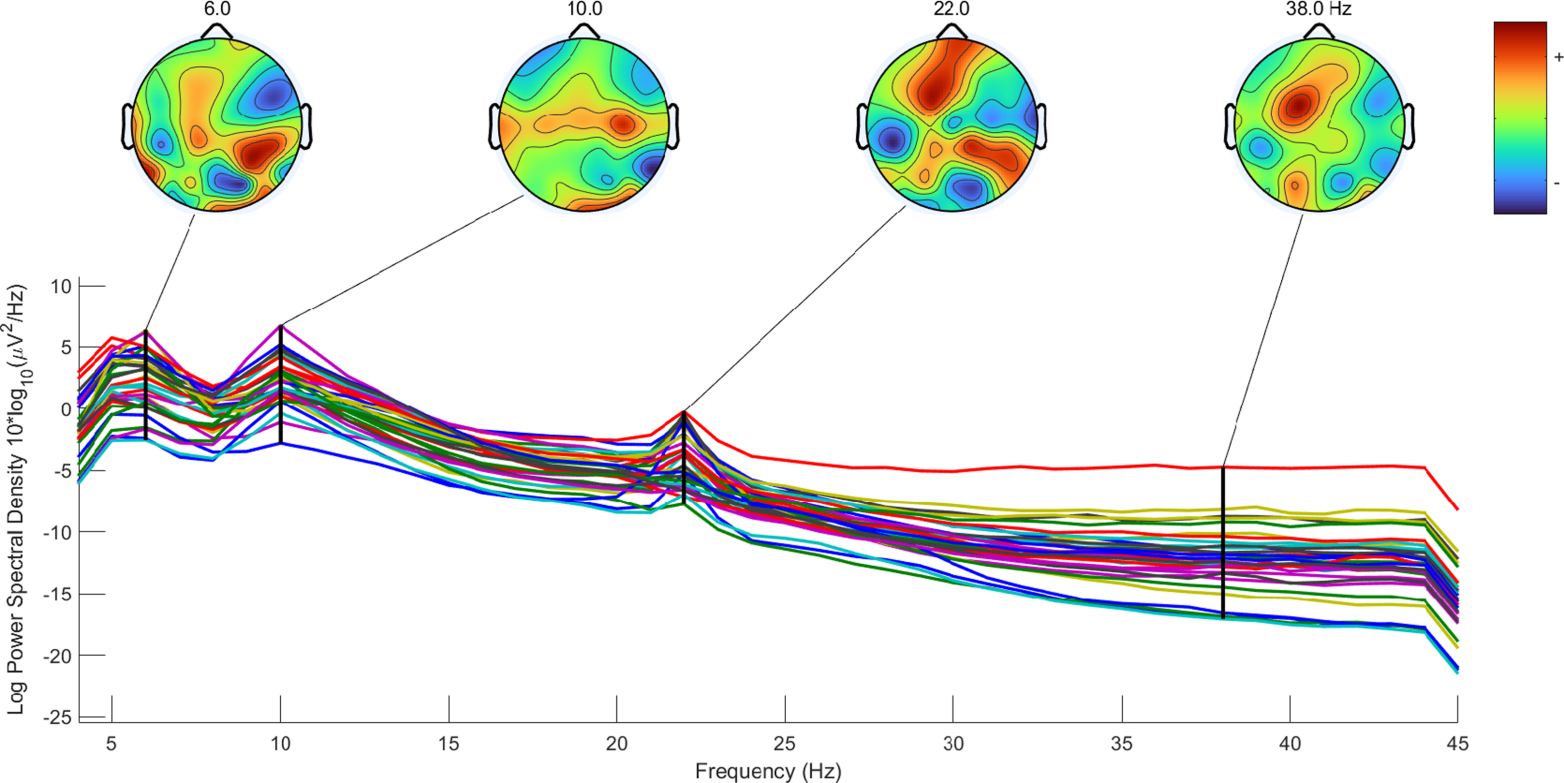
Brain electroencephalogram topography at different frequencies.

In order to clearly show the spatial and frequency characteristics of emotions in EEG, the EEGLAB toolbox was used on the Matlab platform to plot the 32-channel EEG topography of 40 experiments of the first subject in the DEAP dataset at different frequencies, as shown in Figure 11.

In Figure 11, the power distributions across different frequency bands exhibit distinct functional region preferences: *θ* (4–8 Hz) power peaks are primarily concentrated in the right temporal lobe region, suggesting that this frequency band plays a crucial role in emotional regulation and memory encoding; *α* (8–14 Hz) rhythms are predominantly centered in the central-parietal region, reflecting the dynamic balance between sensory integration and attention networks; *β* (14–31 Hz) power enhancement is focused on the central-frontal–parietal region, corresponding to motor preparation and activation of the somatosensory-motor association area; *γ* (>31 Hz), although overall amplitude is low, shows a local increase in the left frontal-central region, indicating rapid synchronized activity during higher-order cognition and multimodal information integration. The above time-frequency-space characteristics are highly consistent with known neural functional areas, proving the physiological rationality and interpretability of this method in emotional and cognitive research.

**Figure 12:**
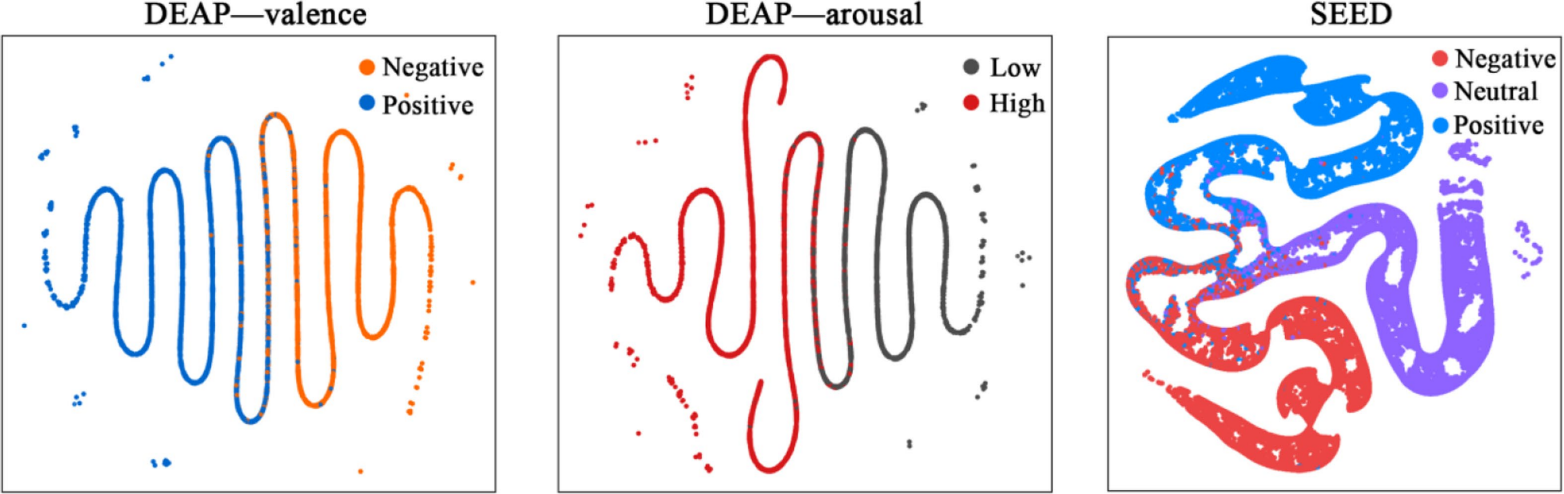
Visualization of classification results.

To visually demonstrate the distinguishability of PH-MTM in EEG features, we extracted high-dimensional features from the classification output layer and used t-distributed stochastic neighborhood embedding (t-SNE) to reduce them to two dimensions, revealing that this layer aggregates all global abstract information after convolution and pooling. The visualization uses all test data from two datasets. Figure 12 shows the EEG classification performance of PH-MTM on the DEAP and SEED datasets.

From Figure 12, we can see that the PH-MTM model can clearly distinguish emotional features, whether it is the binary classification of valence and arousal tasks in the DEAP dataset or the ternary classification in the SEED dataset. This indicates that the model is feasible for identifying emotional features and can achieve good separability.

### 4.5 Ablation experiment

To evaluate the impact of each model module on overall experimental performance across the DEAP dataset, this section employs ablation experiments to progressively dissect the model. By removing key components from PH-MTM, we assess their efficacy and contribution to the sentiment classification task. The baseline model comprises a multi-layer CNN, Dense layer, and single-task learning module. The PH-MTM-SE model, PH-MTM-PHMDense model, and PH-MTM-MTL model, respectively, remove the SE, PHMDense, and MTL modules. By comparing the baseline, models with relevant modules removed, and the complete PH-MTM model, we evaluate each module’s impact on model performance. All models employ identical hyperparameters. As shown in Table 3.

**Table 3:** Performance comparison of different models.

Model	Acc ± Std (%)		*F*1 ± Std (%)		FLOPs (*G*)	Para (*M*)
	Valence	Arousal	Valence	Arousal		
Baseline	95.16 ± 0.30	95.20 ± 0.21	95.11 ± 0.31	95.11 ± 0.21	0.99669516	1.214978
PH-MTM-SE	95.23 ± 0.40	95.66 ± 0.33	95.17 ± 0.40	95.57 ± 0.35	0.99670709	1.215003
PH-MTM-PHMDense	95.40 ± 0.29	95.81 ± 0.16	95.35 ± 0.30	95.73 ± 0.17	0.10333902	**0.821826**
PH-MTM-MTL	94.85 ± 0.33	95.40 ± 0.23	94.80 ± 0.34	95.31 ± 0.24	0.99671576	1.216004
Ours	**95.87 ± 0.31**	**96.21 ± 0.18**	**95.82 ± 0.31**	**96.13 ± 0.19**	**0.10334227**	0.822877

Acc, accuracy; Std, standard deviation; F1, F1-score; FLOPs, floating point operations; Para, parameters; bold values indicate the best performance metrics.

### 4.6 Two-tailed paired *t*-test

Although incorporating the SE, PHMDense, and MTL modules individually into the baseline model yields only marginal performance gains, this does not negate the synergistic effects among the modules. In practice, different modules often complement one another in feature representation, attention mechanisms, and multi-scale information fusion. When combined, their collective contribution may exceed the sum of their individual effects. To quantify this potential synergy, the final model PH-MTM was compared with its ablated variants (PH-MTM-SE, PH-MTM-PHMDense, and PH-MTM-MTL) using a two-tailed paired t-test. Since all models were evaluated on the same test samples, the resulting observations were paired; the normality of the paired differences was verified prior to testing. A *p*-value <0.05 was considered statistically significant. As shown in Table 4, A, B, and C denote the PHMDense, MTL, and SE modules, respectively.

**Table 4:** Two-tailed paired *t*-test for different models.

Model	ΔAcc (*μ* ± *σ*)						Δ*F*1 (*μ* ± *σ*)					
	Valence	*p*	Sig.	Arousal	*p*	Sig.	Valence	*p*	Sig.	Arousal	*p*	Sig.
A + B	0.64 ± 0.21	0.002	*	0.55 ± 0.20	0.003	*	0.65 ± 0.21	0.003	*	0.56 ± 0.20	0.003	*
B + C	0.47 ± 0.21	0.007	*	0.40 ± 0.15	0.004	*	0.47 ± 0.21	0.007	*	0.40 ± 0.15	0.004	*
**A + C**	**1.02 ± 0.15**	**0.000**	*	**0.81 ± 0.23**	**0.001**	*	**1.03 ± 0.15**	**0.000**	*	**0.82 ± 0.23**	**0.001**	*

Acc, accuracy; F1, F1-score; *p*, *p*-value; Sig., significance; **A**, PHMDense; **B**, multi-mask; **C**, SE. *Indicates a statistically significant improvement (*p* < 0.05); bold values indicate the best performance metrics.

The experimental results demonstrate that the combinations *A* + *B* (PHMDense + MTL), *B* + *C* (MTL + SE), and *A* + *C* (PHMDense + SE) all significantly enhance the model’s performance on both valence and arousal tasks. This reflects the complementary interactions between different modules, particularly highlighting the critical importance of inter-module synergistic effects. However, the significant differences among these combinations also reveal variations in the strength of synergistic effects between different modules. PHMDense primarily optimizes computational efficiency by reducing parameters in the Dense layer through matrix decomposition, though it does not directly enhance feature diversity. When combined with MTL, the latter optimizes task-relevant feature representations via robust parameter sharing capabilities, thereby boosting model performance. In contrast, the *B* + *C* (MTL + SE) combination yielded a *p*-value of 0.007, indicating a significant performance improvement albeit to a lesser extent than the *A* + *B* and *A* + *C* combinations. While MTL enhances feature sharing and optimization through hard parameter sharing, the channel re-calibration function of the SE module may not have synergized sufficiently with MTL’s feature learning, resulting in a comparatively modest performance gain. The A + C (PHMDense + SE) combination demonstrated the most pronounced effect, with a p-value of 0.000. PHMDense effectively optimized computational efficiency by reducing network parameters, while SE precisely adjusted feature weights through channel re-calibration, significantly enhancing the model’s performance on both valence and arousal tasks. Their synergistic interaction substantially elevated the model’s overall capability.

### 4.7 Comparative experiment

This section compares the proposed model with other studies in terms of EEG classification performance on the DEAP dataset for binary classification and the SEED dataset for ternary classification, with a focus on evaluating accuracy to comprehensively measure the model’s performance in the field of emotion recognition, as shown in Table 5.

**Table 5:** Performance comparison of the proposed model with other research models.

DEAP				SEED		
Literature	Year	Valence	Arousal	Literature	Year	Accuracy
Raikwar and Mayuri (2025)	2025	94.01%	93.55%	Sidharth et al. (2023a)	2023	93.55%
Zhao et al. (2025)	2025	94.59%	95.21%	Yu et al. (2023)	2023	85.40%
Yin et al. (2021)	2021	90.45%	90.60%	Fernandes et al. (2024)	2024	86.08%
Ghosh et al. (2023)	2023	86.95%	88.87%	Sidharth et al. (2023b)	2023	93.10%
Zhang et al. (2025)	2025	94.22%	92.16%	Wang et al. (2025)	2025	93.14%
Gao et al. (2024)	2024	89.14%	89.65%	Dong et al. (2024)	2024	92.25%
Wang et al. (2024b)	2024	91.92%	92.31%	Shen et al. (2025)	2022	93.19%
Guo et al. (2026)	2026	94.20%	93.90%	Li M. et al. (2023)	2025	94.10%
**Ours**	**2026**	**95.99%**	**96.51%**	**Ours**	**2026**	**94.25%**

Bold values indicate the best performance metrics.

By comparing results with other models on the DEAP and SEED datasets in recent years, the proposed model surpasses current approaches in terms of accuracy for both valence and arousal classification tasks on the DEAP dataset and the three-classification task on the SEED dataset. This further validates the proposed model’s significant advantages in emotion recognition tasks. However, as Table 3 indicates, although PH-MTM achieves excellent performance with accuracy and *F*1-scores of approximately 96% in emotion classification, its improvement over the baseline is not substantial, amounting to only about 1%. This indicates that the potential of EEG in model classification is approaching saturation, and further improvements in model performance require consideration of additional factors or more sophisticated feature fusion methods. Therefore, combining different PPS with EEG is being explored to enhance emotion classification performance.

### 4.8 PH-MTM performance on different peripheral physiological signals

**Figure 13:**
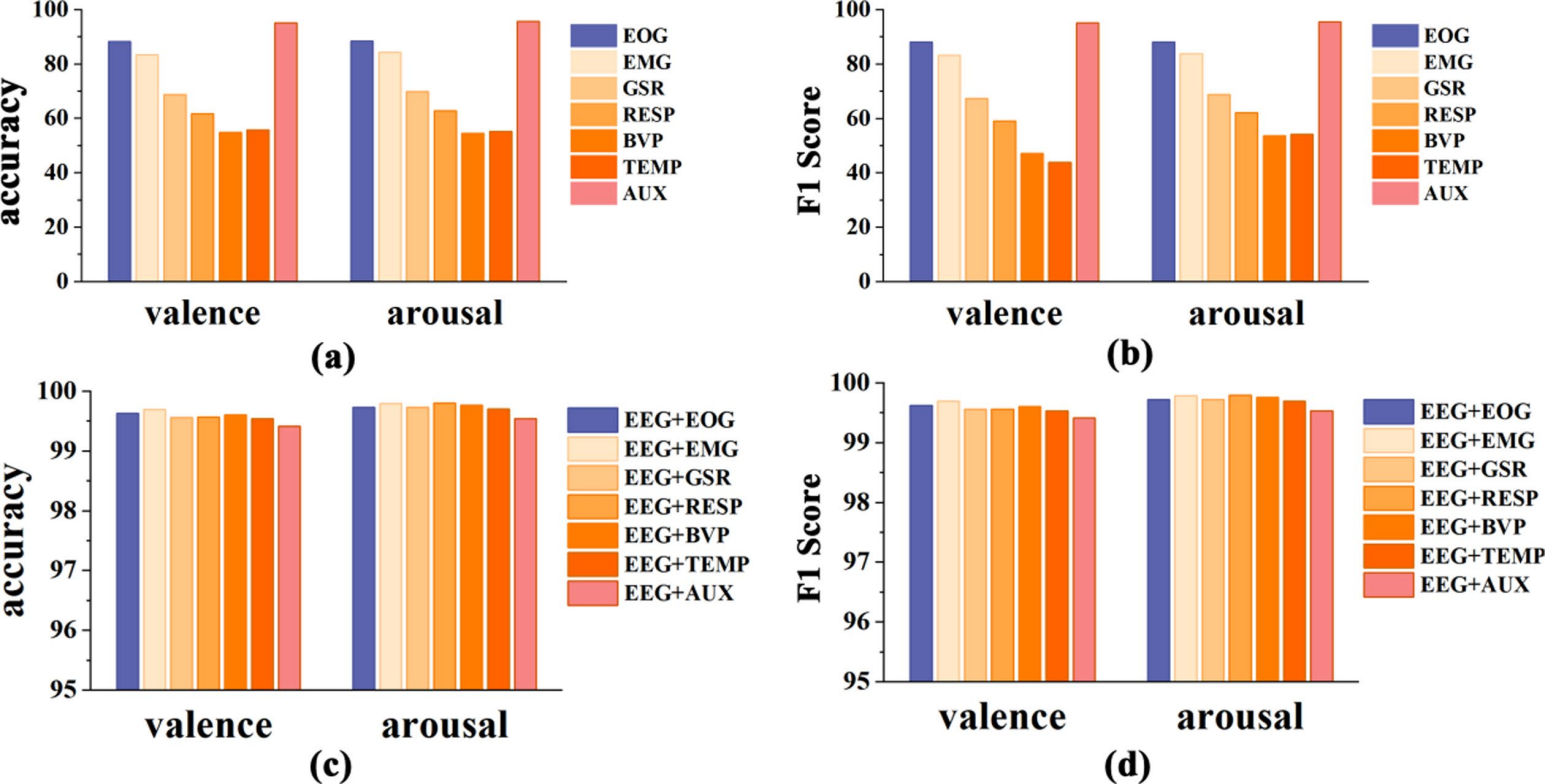
Model performance of the DEAP dataset on the test set. **(a)** Accuracy of single-modality physiological signals, **(b)** F1-score of single-modality physiological signals, **(c)** Accuracy of combined EEG and PPS signals, **(d)** F1-score of combined EEG and PPS signals.

We used 5-fold cross-validation to evaluate the classification performance of different PPS for emotion recognition. The experimental results show that, among single PPS, the EOG signal has the best emotion classification performance, followed by EEG, then EMG, and finally GSR. Under the condition of all peripheral physiological signals being fused, the model’s recognition accuracy and *F*1-score are further improved, classification performance second only to that of EEG, as shown in Table 6. The accuracy and *F*1-score of the single-modality signal test set are shown in Figures 13a,b, respectively.

**Table 6:** Emotion classification performance for single signals.

Model	DEAP-valence			DEAP-arousal		
	Loss ± Std	Acc ± Std (%)	*F*1 ± Std (%)	Loss ± Std	Acc ± Std (%)	*F*1 ± Std (%)
EEG	**0.1127 ± 0.0063**	**95.87 ± 0.31**	**95.82 ± 0.31**	**0.1066 ± 0.0063**	**96.21 ± 0.18**	**96.13 ± 0.19**
EOG	0.2844 ± 0.0072	87.93 ± 0.46	87.79 ± 0.44	0.2879 ± 0.0050	87.76 ± 0.20	87.53 ± 0.27
EMG	0.3655 ± 0.0181	83.46 ± 1.18	83.25 ± 1.20	0.3665 ± 0.0158	83.53 ± 0.96	83.22 ± 0.98
GSR	0.5833 ± 0.0084	68.34 ± 0.74	66.69 ± 0.96	0.5710 ± 0.0088	68.74 ± 0.74	67.92 ± 0.79
RESP	0.6315 ± 0.0065	61.50 ± 0.49	58.20 ± 0.91	0.6282 ± 0.0073	61.47 ± 0.72	60.99 ± 0.71
BVP	0.6834 ± 0.0017	55.46 ± 0.53	42.34 ± 3.29	0.6863 ± 0.0021	54.21 ± 1.10	53.25 ± 0.63
TEMP	0.6830 ± 0.0025	55.99 ± 0.53	45.74 ± 1.90	0.6865 ± 0.0024	54.06 ± 0.89	52.45 ± 1.69
PPS	0.1340 ± 0.0040	95.54 ± 0.20	95.48 ± 0.20	0.1328 ± 0.0045	95.71 ± 0.09	95.62 ± 0.09

Acc, accuracy; Std, standard deviation; *F*1, *F*1-score; EEG, electroencephalogram; EOG, electrooculogram; EMG, electromyogram; GSR, galvanic skin response; RESP, respiration; BVP, blood volume pulse; TEMP, temperature; PPS, peripheral physiological signals; bold values indicate the best performance metrics.

### 4.9 Performance of PH-MTM under different combinations of peripheral physiological signals

By comparing results with other models on the DEAP and SEED datasets in recent years, the proposed model surpasses current approaches in terms of accuracy for both valence and arousal classification tasks on the DEAP dataset and the three-classification task on the SEED dataset. This further validates the proposed model’s significant advantages in emotion recognition tasks. However, as Table 3 indicates, although PH-MTM achieves excellent performance with accuracy and *F*1-scores of approximately 96% in emotion classification, its improvement over the baseline is not substantial, amounting to only about 1%. This indicates that the potential of EEG in model classification is approaching saturation, and further improvements in model performance require consideration of additional factors or more sophisticated feature fusion methods. Therefore, combining different PPS with EEG is being explored to enhance emotion classification performance. The experimental results are shown in Table 7.

**Table 7:** Performance of emotion classification using EEG and peripheral physiological signals.

Model	DEAP-valence			DEAP-arousal		
	Loss ± Std	Acc ± Std (%)	*F*1 ± Std (%)	Loss ± Std	Acc ± Std (%)	*F*1 ± Std (%)
EEG + EOG	0.0134 ± 0.0027	99.65 ± 0.06	99.64 ± 0.06	0.0122 ± 0.0024	99.67 ± 0.04	99.66 ± 0.04
EEG + EMG	**0.0102 ± 0.0005**	**99.69 ± 0.02**	99.69 ± 0.02	**0.0087 ± 0.0020**	**99.74 ± 0.05**	**99.74 ± 0.05**
EEG + GSR	0.0138 ± 0.0013	99.55 ± 0.05	99.54 ± 0.05	0.0103 ± 0.0016	99.67 ± 0.03	99.66 ± 0.03
EEG + RESP	0.0140 ± 0.0009	99.55 ± 0.02	**99.70 ± 0.03**	0.0096 ± 0.0016	99.54 ± 0.02	99.70 ± 0.03
EEG + BVP	0.0146 ± 0.0026	99.53 ± 0.10	99.53 ± 0.10	0.0100 ± 0.0022	99.67 ± 0.09	99.66 ± 0.09
EEG + TEMP	0.0164 ± 0.0013	99.46 ± 0.04	99.46 ± 0.04	0.0123 ± 0.0016	99.61 ± 0.04	99.60 ± 0.04
EEG + PPS	0.0227 ± 0.0024	99.36 ± 0.08	99.35 ± 0.08	0.0227 ± 0.0024	99.39 ± 0.05	99.38 ± 0.05

EEG, electroencephalogram; EOG, electrooculogram; EMG, electromyogram; GSR, galvanic skin response; Resp, respiration; BVP, blood volume pulse; Temp, temperature; PPS, peripheral physiological signals; bold values indicate the best performance metrics.

The model extracted DE and PSD features from different channels and different frequency ranges within the same frequency band for each physiological signal. Although the fusion strategy of EEG and PPS provided the model with more feature dimensions, its effectiveness was not optimal. This may be because the fusion process involves a high degree of information redundancy between different signals, leading to information overload during feature extraction and resulting in slightly poorer model performance in emotion classification tasks. Compared with EEG and other single PPS combinations, the accuracy and *F*1-score of the EEG + PPS combination are slightly lower, and its performance in valence and arousal tasks has not been significantly improved. Therefore, although the fusion of EEG and PPS signals can provide more feature information, its actual effectiveness is limited by signal redundancy and feature fusion strategies. In fact, only by selecting appropriate physiological signals for fusion can the accuracy and performance of emotion assessment be effectively improved. The accuracy and *F*1-scores of the test set for different combinations of EEG and PPS are shown in Figures 13c,d, respectively.

As can be seen from Figure 13, in terms of accuracy and *F*1-score, all combinations of PPS have outperformed EEG, which is commonly used in the field of emotion recognition, in emotion classification. This provides new ideas for follow-up research. Furthermore, combining different physiological signals with EEG can effectively improve the classification performance of the model.

### 4.10 Cost analysis

Currently, most emotion recognition research focuses on improving accuracy and *F*1-scores, while relatively few studies optimize model complexity metrics such as FLOPs and parameters. In contrast, this model is designed not only to enhance performance but also incorporates the novel PHMDense architecture to reduce computational costs and minimize model parameters, thereby expanding possibilities for subsequent application deployment. This section compares the computational cost of PH-MTM with other deep neural networks for physiological signal-based emotion recognition developed in recent years, as shown in Table 8. The results demonstrate that PH-MTM possesses fewer parameters (0.82 million) and achieves higher accuracy compared to other models.

**Table 8:** Performance comparison between PH-MTM and other state-of-the-art methods on the DEAP dataset.

References	Time	Modal	Feature	Model	Target class	Acc ± Std (%)		*F*1 ± Std (%)	
						Valence	Arousal	Valence	Arousal
Zhang et al. (2024)	2024	EEG	DE	TPRO-NET	2	97.87 ± 1.89	98.08 ± 1.83	97.78 ± 1.91	97.89 ± 1.90
					8	97.63 ± 2.38	97.74 ± 2.26	97.78 ± 1.91	97.89 ± 1.90
Ju et al. (2024)	2024	EEG	DE	TDMNN	3	98.08 ± 2.13	98.25 ± 2.85	—	—
Kim et al. (2022)	2022	EEG	Raw signal	CNN-BN	2	99.10 ± 0.10	99.48 ± 0.07	99.09 ± 0.10	99.47 ± 0.07
Xu et al. (2023)	2023	EEG	DE, PSD	AMDET	2	97.48 ± 0.99	96.85 ± 1.66	—	—
**Ours**	**2025**	**EEG + EMG**	**DE, PSD**	**PH-MTM**	**2**	**99.69 ± 0.02**	**99.69 ± 0.02**	**99.74 ± 0.05**	**99.74 ± 0.05**

EEG, electroencephalogram; EMG, electromyogram; DE, differential entropy; PSD, power spectral density; Acc, accuracy; Std, standard deviation; F1, F1-score; TPRO-NET, temporal projection network; TDMNN, temporal-difference minimizing neural network; CNN-BN, 3D convolutional neural network with channel bottleneck; AMDET, attention-based multiple dimensions; EEG, transformer; PH-MTM, parametric hypermatrix multi-task multi-modal emotion recognition model; bold values indicate the best performance metrics.

The results show that the PH-MTM model significantly outperforms other comparison models in terms of classification performance. This finding indicates that combining appropriate PPS with EEG can effectively compensate for the limitations of single EEG modality in emotion representation, thereby significantly improving the accuracy of emotion recognition. PPS can reflect changes in ANS activity, complementing the central nervous system dynamics revealed by EEG, enabling the model to capture more comprehensive emotional characteristics from a multidimensional physiological perspective. Therefore, PH-MTM not only demonstrates significant advantages in multimodal physiological signal emotion recognition but also provides a feasible path and theoretical support for constructing high-precision emotion recognition systems.

## 5 Discussion

### 5.1 Visualization of the distinguishability of EEG features across different frequency bands

**Figure 14:**
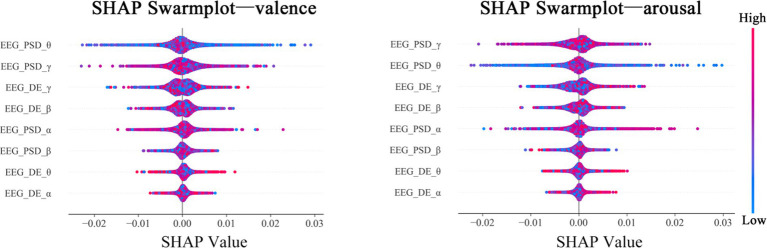
Visualization of two-class features for the Valence and Arousal tasks in the DEAP dataset.

**Figure 15:**
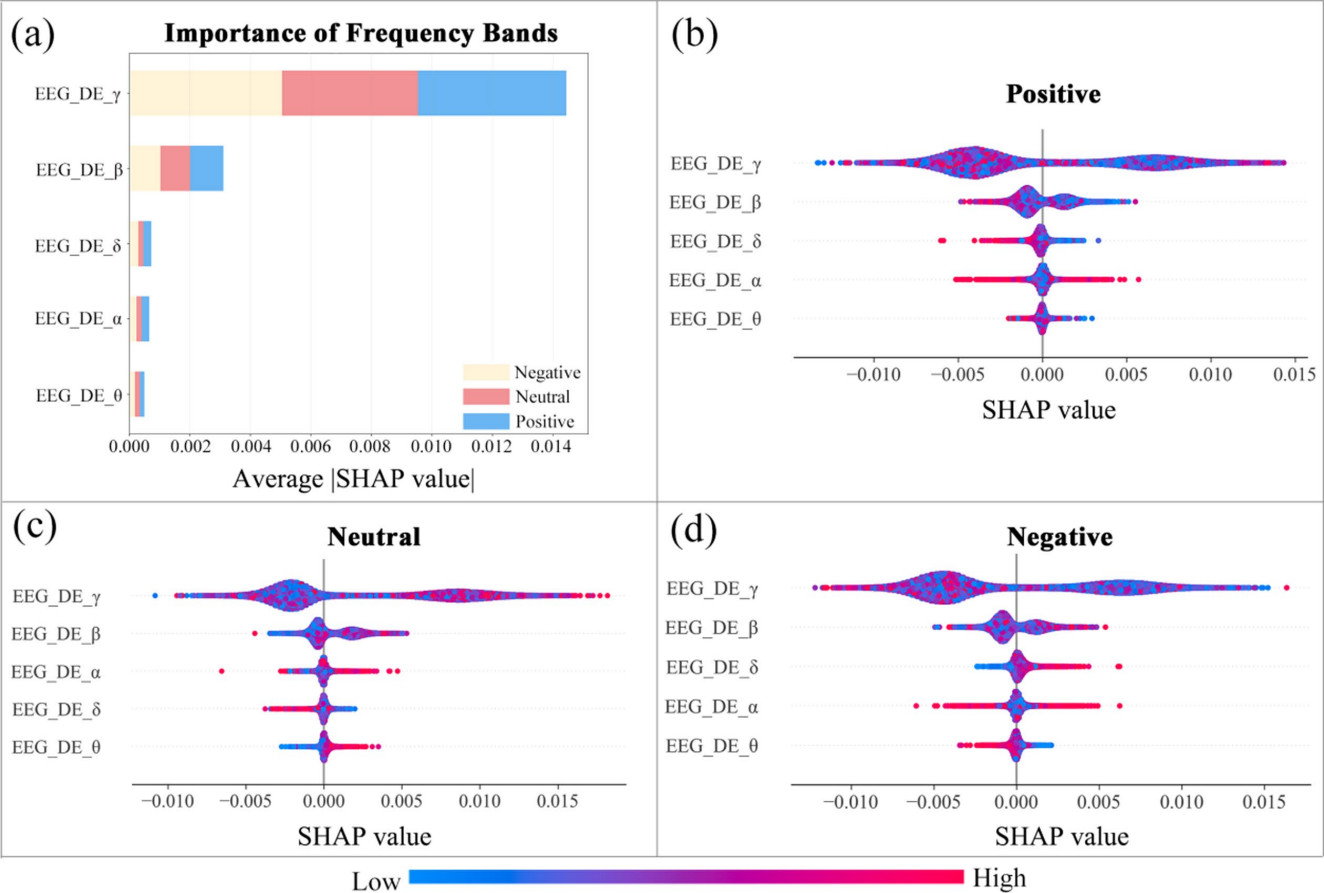
Visualization of three-class features in the SEED dataset. **(a)** Shows the average absolute SHAP values of different frequency bands in the three-class classification task. Panels **(b–d)** use bee swarm plots to further present the SHAP values for the positive, neutral, and negative classifications, thereby highlighting the importance of each frequency band feature in emotion classification.

Due to the black-box nature of deep learning models, their internal decision-making processes are difficult to explain directly, making it challenging to understand the feature contributions in emotion classification. To reveal the model’s dependence on different EEG frequency bands, we employed the SHAP method for visualization analysis. SHAP is a game theory-based model interpretation method that provides transparent global and local explanations for complex machine learning models (Lundberg and Lee, 2017). Its advantage lies in accurately allocating the contribution of each feature to the model prediction, making the model decision-making process more interpretable and consistent. In the DEAP dataset, the test set comprises 30,720 samples, corresponding to a binary classification task of Valence and Arousal. In the SEED dataset, the test set contains 60,968 samples, corresponding to a three-class classification task of positive, neutral, and negative emotions. Based on the test samples from these two datasets, we conducted SHAP-based feature visualization analyses, and the results are presented in Figures 14 and 15. This enables a more intuitive observation of the importance distribution of different frequency band features in binary and three-class emotion recognition tasks, thereby providing a basis for subsequent feature selection and model optimization.

As can be seen from the figure above, the EEG *θ* and *γ* band features exhibit the greatest contribution in the valence and arousal classification tasks respectively, with their SHAP values demonstrating stable and significant influence across both tasks. Furthermore, the PSD features generally perform better than the DE features, proving more effective at capturing spectral information from brain signals and making a greater and more stable contribution to emotion classification.

As shown in the above figure, the EEG *γ* band makes the greatest contribution to the emotion classification task in the SEED dataset (including positive, neutral, and negative emotions). Particularly, the SHAP values of the *γ* band exhibit stable and significant positive influences across the three emotional states. In contrast, other frequency bands such as *β*, *α*, *δ*, and *θ* show smaller contributions, with varying impacts across different emotion classifications. In the positive emotion classification, the SHAP values of the α band predominantly show negative influence, while the γ band plays an important role in both positive and negative emotion classifications, highlighting its key role in emotion classification.

### 5.2 Visualization of feature discriminability in multimodal embeddings

**Figure 16:**
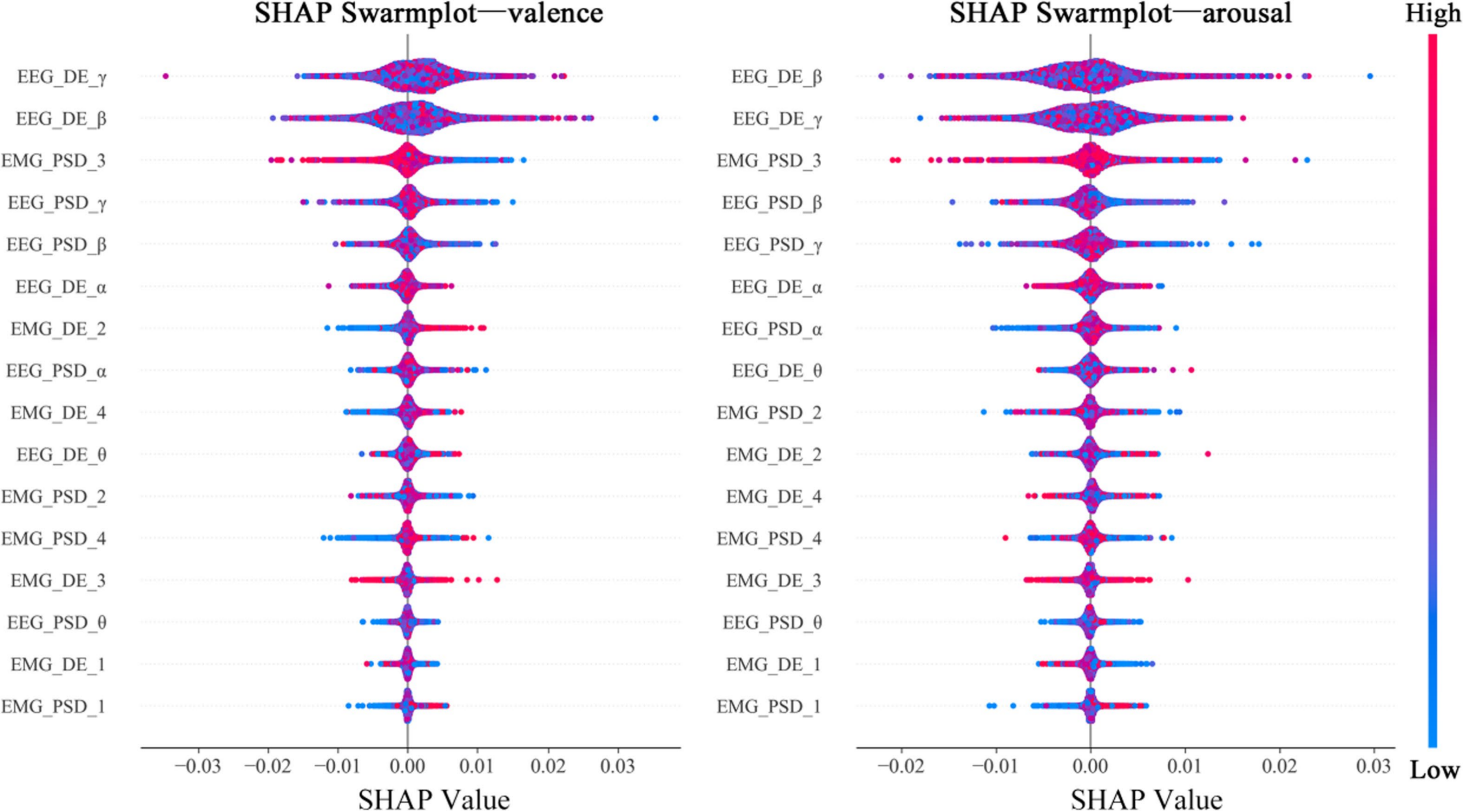
Visualization of EEG + EMG features in the DEAP dataset.

As shown in Table 7, the combination of EEG and EMG in the DEAP dataset performs best. Therefore, in this section, we visualize the feature contribution of each corresponding frequency band of EEG and EMG to emotion classification, aiming to reveal the role of each frequency band in emotion recognition and the contribution of EEG and EMG to model prediction, thereby providing new ideas for subsequent researchers in signal combination and frequency band fusion methods, as shown in Figure 16.

Based on the results from Figure 16, EEG serves as the Khare and Bajaj, 2020 primary modality for emotion classification in both the Valence and Arousal tasks, while EMG functions as an auxiliary modality. This finding aligns with research in the field of emotion recognition (Chen et al., 2026a; Yuvaraj et al., 2023; Cheng et al., 2020; Khare and Bajaj, 2020; Liu et al., 2021; Li et al., 2018; Guo et al., 2022; Zhang et al., 2021), where the majority of studies focus on EEG features, as EEG effectively reflects an individual’s emotional state. In contrast, EMG primarily reflects muscle activity and, although it has some influence on emotional states, is typically used as an auxiliary signal in emotion classification tasks, contributing less. Furthermore, the *γ* and *β* bands in the DE and PSD features of EEG make significant contributions to emotion classification tasks. The high-frequency bands of EMG, compared to the low-frequency bands, have a greater contribution to emotion classification.

Overall, the SHAP visualizations presented above clearly show that the *γ* and *β* bands of EEG contribute significantly to emotion classification compared to other frequency bands, a result consistent with most studies in the field of emotion recognition (Lee et al., 2026; Bilgin and Mert, 2024; Lin et al., 2023; Soundariya and Thangaraj, 2026). Additionally, although EMG signals play a less prominent role in emotion recognition compared to EEG, they still provide valuable information as an auxiliary signal. In the future, multimodal emotion recognition methods combining EEG and EMG could further enhance the accuracy and robustness of emotion classification, particularly in handling complex and dynamic emotional states. This study provides important insights for subsequent researchers, particularly in selecting appropriate features and signal modalities, which can help optimize the design of emotion recognition systems and promote the application of emotion computing technologies in areas such as brain-computer interfaces and human-computer interaction.

### 5.3 Cost calculation analysis

The computational cost of PH-MTM was compared with that of other deep neural networks related to physiological signal emotion recognition over the past 5 years, as shown in Table 9. The results indicate that PH-MTM has fewer parameters (0.82 M) and higher accuracy compared to other models.

**Table 9:** Comparison of PH-MTM with other literature calculation costs.

Modal	References	Feature	Acc ± Std (%)		FLOPS (*G*)	Params (*M*)
			Valence	Arousal		
TPRO-NET	Wang et al. (2024b)	EEG	97.87 ± 1.89	98.08 ± 1.83	**9.11 × 10** ^**−2**^	1.10 × 10^0^
TDMNN	Shen et al. (2025)	EEG	98.08 ± 2.13	98.25 ± 2.85	3.12 × 10^4^	1.53 × 10^1^
CNN-BN	Guo et al. (2026)	EEG	99.10 ± 0.10	99.48 ± 0.07	2.27 × 10^1^	1.11 × 10^0^
AMDET	Li M. et al. (2023)	EEG	97.48 ± 0.99	96.85 ± 1.66	0.30 × 10^−1^	0.30 × 10^0^
**PH-MTM**	**Ours**	**EEG + EMG**	**99.69 ± 0.02**	**99.74 ± 0.05**	**1.03 × 10** ^**−1**^	**8.21 × 10** ^**−1**^

Acc, accuracy; Std, standard deviation; FLOPs, floating point operations; Para, parameters; TPRO-NET, temporal projection network; TDMNN, temporal-difference minimizing neural network; CNN-BN, 3D convolutional neural network with channel bottleneck; AMDET, attention-based multiple dimensions; EEG, transformer; PH-MTM, parametric hypermatrix multitask multi-modal emotion recognition model; bold values indicate the best performance metrics.

In terms of cost calculation, although PH-MTM’s FLOPS is 1.03 × 10^−1^ G, slightly higher than TPRO-NET, it is still considered lightweight compared to other models. At the same time, it achieves the best recognition accuracy with the fewest parameters (8.21 × 10^−1^ M), fully demonstrating the advantages of the model and providing new ideas for subsequent lightweight model design.

## 6 Conclusion

In this paper, we propose a parameterized hypermatrix multi-task multimodal emotion recognition model based on the fusion of EEG and PPS. First, we pre-process EEG and PPS using the DEAP public dataset, extract DE and PSD features, and map EEG features to a “band number × 8 × 9” grid structure based on the 10–20 system. Then, the improved SE attention mechanism is used to adaptively allocate frequency band feature weights, and combined with CNN to deeply fuse cross-frequency band and cross-channel features. Finally, a lightweight custom Dense layer based on matrix decomposition and a multi-task learning framework are adopted to improve the accuracy and efficiency of emotion classification.

The proposed method achieved accuracy rates of 99.69 and 99.74% on the DEAP dataset for the valence and arousal tasks, respectively, validating the model’s efficiency and stability. To further validate the effectiveness of the frequency band fusion method, we conducted experiments on 62-channel EEG data from the SEED dataset. The test set accuracy reached 94.25%, proving the effectiveness of the method and breaking through the limitations of traditional 32-channel EEG. Additionally, the combined classification performance of all PPS was superior to that of individual EEG. This method fully demonstrates the effectiveness of multimodal physiological signal fusion strategies. In addition, the new Dense layer based on matrix decomposition effectively reduces the number of model parameters, providing new ideas for the subsequent development of low-cost, more efficient multimodal emotion recognition systems. However, the current experiment was conducted on offline data and has not yet been fully verified in terms of performance and stability in low-latency, real-time emotion recognition scenarios. Future research will focus on optimizing this issue to further improve the model’s generalization performance and computational efficiency.

## Data Availability

The original contributions presented in the study are included in the article/supplementary material, further inquiries can be directed to the corresponding author.
